# Hepatitis C Virus RNA Replication Depends on Specific *Cis-* and *Trans*-Acting Activities of Viral Nonstructural Proteins

**DOI:** 10.1371/journal.ppat.1004817

**Published:** 2015-04-13

**Authors:** Teymur Kazakov, Feng Yang, Harish N. Ramanathan, Andrew Kohlway, Michael S. Diamond, Brett D. Lindenbach

**Affiliations:** 1 Department of Microbial Pathogenesis, Yale University School of Medicine, New Haven, Connecticut, United States of America; 2 Department of Molecular Biophysics and Biochemistry, Yale University, New Haven, Connecticut, United States of America; 3 Departments of Medicine, Molecular Microbiology, and Pathology & Immunology, Washington University School of Medicine, St. Louis, Missouri, United States of America; The Scripps Research Institute, UNITED STATES

## Abstract

Many positive-strand RNA viruses encode genes that can function in *trans*, whereas other genes are required in *cis* for genome replication. The mechanisms underlying *trans-* and *cis-*preferences are not fully understood. Here, we evaluate this concept for hepatitis C virus (HCV), an important cause of chronic liver disease and member of the *Flaviviridae* family. HCV encodes five nonstructural (NS) genes that are required for RNA replication. To date, only two of these genes, NS4B and NS5A, have been *trans*-complemented, leading to suggestions that other replicase genes work only in *cis*. We describe a new quantitative system to measure the *cis*- and *trans*-requirements for HCV NS gene function in RNA replication and identify several lethal mutations in the NS3, NS4A, NS4B, NS5A, and NS5B genes that can be complemented in *trans*, alone or in combination, by expressing the NS3–5B polyprotein from a synthetic mRNA. Although NS5B RNA binding and polymerase activities can be supplied in *trans*, NS5B protein expression was required in *cis*, indicating that NS5B has a *cis*-acting role in replicase assembly distinct from its known enzymatic activity. Furthermore, the RNA binding and NTPase activities of the NS3 helicase domain were required in *cis*, suggesting that these activities play an essential role in RNA template selection. A comprehensive complementation group analysis revealed functional linkages between NS3-4A and NS4B and between NS5B and the upstream NS3–5A genes. Finally, NS5B polymerase activity segregated with a daclatasvir-sensitive NS5A activity, which could explain the synergy of this antiviral compound with nucleoside analogs in patients. Together, these studies define several new aspects of HCV replicase structure-function, help to explain the potency of HCV-specific combination therapies, and provide an experimental framework for the study of *cis*- and *trans*-acting activities in positive-strand RNA virus replication more generally.

## Author Summary

RNA viruses encode one or more proteins that form an RNA replicase, which serves to propagate the viral RNA genome. Some replication genes can function in *trans*, meaning that proteins expressed by one viral genome can complement and assist in the replication of a defective mutant virus within the same cell. Other viral genes work in *cis*, meaning that they are unable to complement defective mutants; these *cis*-acting genes therefore link the replication of a viral RNA genome to its translation. Because genetic complementation reflects the formation of molecular interactions that were previously absent or nonfunctional, understanding the *cis*- and *trans*-activities of viral replication proteins can provide insights into the structure and function of viral replicases. Here we develop a novel methodology to study interactions between components of the viral replicase to define essential *cis*- and *trans*-activities for all five hepatitis C virus (HCV) replication gene products. Our studies show that representative defects in each HCV replication protein can be complemented in *trans*. However, specific features of NS3 and NS5B were required in *cis*, indicating that these proteins specifically function to replicate the genome that encodes them. These data define important activities of the HCV replication proteins and provide new insight into how these proteins function together as an enzyme complex.

## Introduction

The low-fidelity of RNA virus replication gives rise to swarms of mutant variants [1], which can genetically interact with one another. Mutant viruses may complement or inhibit one another in *trans*, with important consequences on virus evolution, pathogenesis, and the emergence of drug-resistance [2–5]. For instance, subgenomic (i.e. less than genome length) defective-interfering (DI) viruses are dependent on, and compete with, helper viruses with intact genomes [6]. Because of this competition, many positive-strand RNA viruses have evolved genome quality control mechanisms that require specific viral genes in *cis* [7–14]. In some cases, essential *cis*-acting RNA sequences or secondary structures are embedded within the viral coding region [15–19]. In other cases, RNA replication requires ribosomal translation through a specific gene region, although the gene product is not necessarily required in *cis* [7,11,20]. Finally, some *cis*-acting viral proteins recruit the RNA from which they were translated into RNA replication complexes [9–11,21].

**Fig 1:**
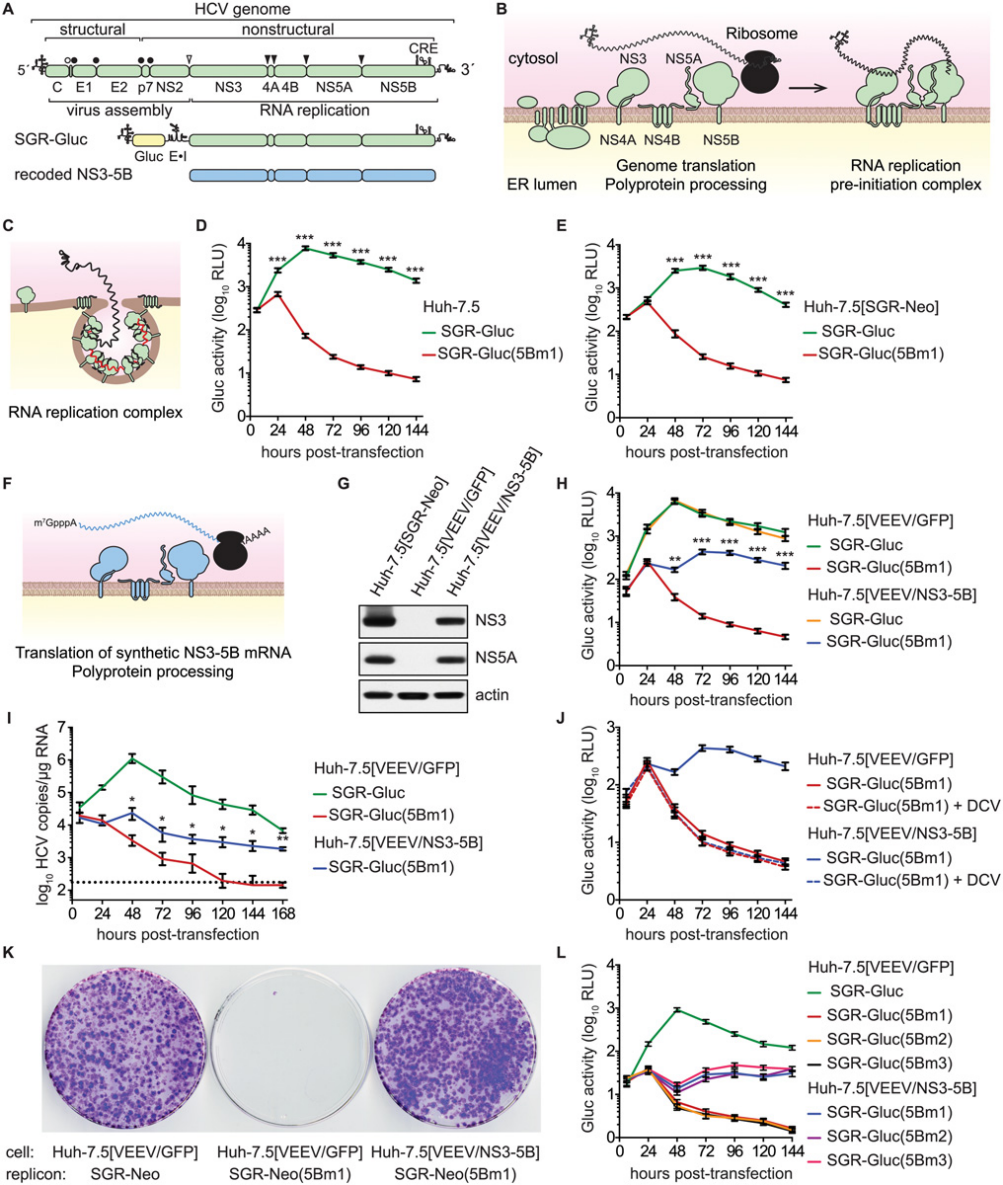
HCV NS5B can be complemented in *trans*. (**A**) Overview of the HCV genome, SGR-Gluc, and the recoded NS3–5B region; CRE, cis-responsive element; E•I, encephalomyocarditis virus IRES. (**B**) Model of HCV genome translation, polyprotein processing, and recruitment of the genome out of translation and into a proto-replication complex. (**C**) Model of an active, membrane-bound HCV replication complex; red line, negative-strand RNA (**D**) HCV replication-dependent expression of Gluc activity. Media were collected at the indicated times post-transfection and assayed as described in Materials and Methods. Values represent mean ± SD from transfections done in triplicate and normalized to untransfected controls; ***, p < 0.001 by Student's *t*-test. This experiment was performed >12 times with similar results. (**E**) SGR-Gluc(5Bm1) does not replicate in Huh-7.5[SGR-Neo] cells. Values represent mean ± SD from transfections done in triplicate and normalized to untransfected controls; ***, p < 0.001 by Student's *t*-test. This experiment was repeated four times with similar results. (**F**) Model of NS3–5B expression from a recoded mRNA. This mRNA is not replicated because it lacks 5', 3', and internal CREs of the HCV genome. (**G**) Western blot of NS3 (antibody 9G2), NS5A (antibody 9E10), and actin (antibody AC-15) in lysates prepared from the indicated cell lines. (**H**) SGR-Gluc(5Bm1) replicates in Huh-7.5[VEEV/NS3–5B] cells but not in Huh-7.5[VEEV/GFP] cells. Values represent mean ± SD from transfections done in triplicate and normalized to untransfected controls; **, *p* < 0.01; ***, p < 0.001 by Student's *t*-test of SGR-Gluc(5Bm1) in Huh-7.5[VEEV/NS3–5B] vs. Huh-7.5[VEEV/GFP] cells. This experiment was performed eight times with similar results. (**I**) HCV RNA copies were determined by RT-qPCR. Values represent mean ± SD from transfections performed in triplicate and normalized to untransfected controls. This experiment was repeated three times with similar results. Dotted line, limit of detection for the qRT-PCR assay; *, *p* < 0.05; **, *p* < 0.01 by Students *t*-test of SGR-Gluc(5Bm1) RNA values in Huh-7.5[VEEV/NS3–5B] vs. Huh-7.5[VEEV/GFP] cells. (**J**) Complementation of SGR-Gluc(5Bm1) is sensitive to DCV (dashed lines). For comparison, the untreated SGR-Gluc(mut5B) samples, which were performed in parallel, are reproduced from panel D. Values represent mean ± SD from transfections performed in triplicate and normalized to untransfected controls. This experiment was repeated three times with similar results. (**K**) G418-resistant colony formation by SGR-Neo or SGR-Neo(5Bm1) was determined in the indicated cell lines by crystal violet staining. (**L**) Complementation of SGR-Gluc(5Bm1), SGR-Gluc(5Bm2), and SGR-Gluc(5Bm3) in Huh-7.5[VEEV/NS3–5B] cells. Values represent mean ± SD from transfections done in triplicate and normalized to untransfected controls. This experiment was performed three times with similar results.

One group of viruses exhibiting marked *cis*-preferences is the *Flaviviridae*, a large family of enveloped, positive-strand RNA viruses that includes several globally important human and animal pathogens [22]. Members of this family are genetically diverse but share similarities in genome organization, mechanisms of gene expression, and replication strategies. As a model of RNA replication, the best-characterized member of this family is hepatitis C virus (HCV), a human pathogen that causes chronic liver disease, cirrhosis, and liver cancer, and is a major target of antiviral design [23]. The 9.6-kb HCV genome lacks a 5' cap and 3' polyadenylated tail but has highly structured 5' and 3' non-coding regions, as well as an internal RNA *cis*-replication element (CRE) within the coding region (Fig 1A). These RNA structures are important *cis*-acting sequences that direct translation and replication of the viral genome, including a 5' internal ribosome entry site (IRES) to initiate cap-independent translation.

As for all positive-strand RNA viruses, the HCV genome is translated directly, producing a polyprotein that is cleaved by cellular and viral proteinases into structural proteins—core, E1, and E2—and nonstructural (NS) proteins—p7, NS2, NS3, NS4A, NS4B, NS5A, and NS5B (Fig 1A and 1B). After multiple rounds of translation, a subset of NS proteins then recruits the genome out of translation and into a membrane-bound replication complex (replicase) that contains multiple copies of NS3–5B (Fig 1B and 1C). The core–NS2 genes are involved in virus particle assembly but are dispensable for RNA replication [24]; in contrast, NS3–5B are all required for RNA replication and is the minimal set of HCV genes necessary to produce a self-replicating subgenomic RNA replicon (SGR) [25]. RNA replication is thought to occur via *de novo* (i.e., unprimed) initiation at or near the 3' end of the template genome, production of a complementary negative strand, and iterative use of the negative strand as a secondary template to make many new positive-strand genomes [26,27].

Much is known about the structure and function of individual NS3–5B proteins, although the molecular architecture of the replicase is not yet known. NS3 is a bifunctional enzyme, encoding an N-terminal serine protease domain and a C-terminal helicase domain. The serine protease requires interaction with NS4A for optimal activity and is responsible for cleaving the downstream polyprotein [28]; it also proteolytically inactivates specific cellular substrates including MAVS [29,30], TRIF [31], DDB1 [32], and GPx8 [33]. The C-terminal RNA helicase/NTPase activities of NS3 are essential for RNA replication [34,35], although the specific roles of these activities are unknown. For instance, this domain can unwind double-stranded RNA and DNA *in vitro* [36], but direct evidence is lacking that it binds to or unwinds viral RNA during the replication cycle *in vivo*. The small NS4A protein binds to NS3, acts as a cofactor for both NS3 activities [28,37], and anchors the NS3-4A heterodimer in the membrane [38]. NS4B is a polytopic membrane protein that can oligomerize and may serve as a scaffold for replicase assembly [39,40]. NS5A is an RNA-binding phosphoprotein that contains an N-terminal amphipathic membrane anchor, a structured zinc-binding domain that directs NS5A homodimerization in either of two alternate conformations, and a large, natively unfolded C-terminal domain [41]. The precise role of NS5A during RNA replication is not yet clear, but it is known to recruit cellular factors essential for replicase assembly and function, including cyclophilin A [42–46], phosphatidylinositol 4-kinase IIIα [47], and the vesicle trafficking protein VAP-A [48–50]. NS5B is the viral RNA-dependent RNA polymerase and has a C-terminal membrane tail anchor [51,52]. The NS3 serine protease, NS5B polymerase, and NS5A proteins are the major targets of HCV-specific antiviral compounds [23].

Mutations in the HCV core-NS2 genes, including large in-frame deletions, can be complemented in *trans* [53–56]. However, among the genes required for RNA replication, only NS4B and NS5A have been shown to be *trans*-complemented [57–62]. These observations have led to the suggestion that the other NS genes work only in *cis*, although the underlying basis of these *cis* requirements remains unclear. Here, we describe newly developed quantitative tools to study the *cis*- and *trans*-requirements of HCV RNA replication and identify loss-of-function mutations in NS3, NS4A, and NS5B that can be complemented in *trans*. Whereas NS5B RNA binding and polymerase activities could be supplied in *trans*, NS5B expression was required in *cis*, suggesting that NS5B has a structural role in replicase assembly. A subset of NS3 activities was required in *cis*, including the helicase RNA binding and NTPase active site residues, implicating these helicase activities in the recruitment of an RNA template for replication. Complementation group analysis indicated that NS3-4A and NS4B activities are coordinated, whereas NS5B activity requires an intact set of upstream NS3-5A genes. We followed up on this latter observation by showing that NS5B segregates with a daclatasvir-sensitive activity of NS5A. These results reveal new aspects of HCV replicase structure-function, provide insights into combination anti-HCV therapy, and establish an experimental platform to study the genetic and functional interactions of gene products from positive-strand RNA viruses.

## Results

### NS5B can be supplied in *trans*

HCV SGRs encoding reporter genes provide a quantitative readout of HCV RNA replication [63,64]. To monitor HCV replication levels over time, we used SGR-Gluc (Fig 1A), an HCV genotype 2a (strain JFH-1) SGR that autonomously replicates and expresses the *Gaussia princeps* luciferase (Gluc) [65]. At early times post-transfection of Huh-7.5 hepatoma cells with SGR-Gluc RNA transcripts, Gluc expression increased and reached maximal expression by 48 hours (Fig 1D); the decline in Gluc activity at later times corresponded with the onset of cytopathic effects caused by JFH-1 replication [55,64,66]. In contrast, SGR-Gluc(5Bm1), a mutant replicon containing inactivating point mutations of the Mg^++^-coordinating polymerase active site residues (Table 1), expressed Gluc only at early time points post-transfection (Fig 1D), consistent with translation of the input RNA followed by RNA turnover [65].

**Table 1:** Mutants used in this study.

		Mutations^a^			
Gene	Name	JFH-1	H77	Rationale	References
NS5B	5Bm1	D318N	D2738	NS5B polymerase active site, Mg^++^ coordination	[25,34,67]
		D319N	D2739		
	5Bm2	R168A	R2588	NS5B RNA binding groove	[68,69]
		K172A	K2592		
	5Bm3	As above		Combined 5Bm1 and 5Bm2	This study
	5A*5B	S1	S2421	Nonsense mutation at NS5B start	
	5Brc	n.a.		Recoded NS5B to create -1 ORF	This study
	5Bfs	T40–R592		-1 Frameshift at NS5B codon 40	This study
NS3	3m1	H57A	H1083	Serine protease active site	[70]
	3m2	S139A	S1165	Serine protease active site	[70,71]
	3m3	T269A	T1295	Helicase RNA binding	[72,73]
	3m4	D290A	D1316	Helicase NTPase active site	[34,72,74]
		E291A	D1317		
	3m5	W501A	W1527	Helicase stacking	[73,75–77]
	3m6	ΔF184	F1210	Linker between the NS3 serine protease and helicase domains	[78]
		ΔS185	T1211		
		ΔD186	D1212		
	3m7	P190G	P1216	Linker between the NS3 serine protease and helicase domains	[78]
		P191G	P1217		
NS4A	4Am1	Y45A	Y1702	Essential, conserved Tyr in NS4A C-terminal acidic domain	[65,79]
	4Am2	A11L	A1668	NS4A transmembrane dimerization	[80]
NS4B	4Bm1	E226A	E1937	Determinant of NS4B homotypic interaction and membrane alteration	[40,58]
NS5A	5Am1	S232I	S2204	Determinant of NS5A hyperphosphorylation	[58,81,82]
	DCV^R^	F28S	M2000	Daclatasvir resistance	[59]
NS3–NS5A	3–5Arc	n.a.		Recoded NS3 codon 111 to NS5A codon 120	This study
NS3, NS5B	3&5Bm	As above		Combined 3m6 and 5Bm1	This study
NS4A–NS5A	4A–5Am	As above		Combined 4Am1, 4Bm1, and 5Am1	This study
NS3–NS5B	3–5Bm	As above		Combined 3m6, 4Am1, 4Bm1, 5Am1, and 5Bm1	This study

^a^Residues are numbered by their codon position within each gene of the JFH-1 subgenomic replicon or in the polyprotein of HCV reference strain H77; n.a., not applicable.

We tested whether the replication defect of SGR-Gluc(5Bm1) could be *trans*-complemented in Huh-7.5[SGR-Neo] cells, which stably harbor a functional JFH-1 SGR that confers resistance to G418 [66]. SGR-Gluc efficiently replicated in these cells, whereas SGR-Gluc(5Bm1) did not (Fig 1E). Thus, SGR-Gluc(5Bm1) was not *trans*-complemented in Huh-7.5[SGR-Neo] cells, consistent with prior results indicating that NS5B is not supplied in *trans* by an active replicon [57,61,83]. We hypothesized that active RNA replication competed with complementation, such that NS5B expressed by a replicon might be unavailable to function in *trans*. To test this idea, we expressed NS3–5B outside the context of a functional HCV replicon by using a recoded 6015-nt NS3–5B gene region containing 1379 silent mutations (Fig 1A, 1F, and S1 Text). The rationale for recoding HCV NS3–5B was to: 1) ensure efficient expression by using preferred human codons; 2) minimize RNA secondary structures, including known and potential *cis*-acting replication elements within the NS3–5B coding region; and 3) allow for selective amplification of HCV replicons vs. synthetic NS3–5B genes during downstream analysis (e.g. RT-PCR). The recoded NS3–5B region was expressed in Huh-7.5 cells by using a noncytopathic SGR derived from Venezuelan equine encephalitis virus (VEEV), a member of the *Alphavirus* genus of the *Togaviridae* family of positive-strand RNA viruses. This expression vector was chosen because noncytopathic alphavirus vectors: 1) stably and abundantly express foreign genes [84,85]; 2) accommodate large insertions [86]; and 3) have been used successfully in *trans*-complementation studies with members of the *Flaviviridae*, including HCV [86–89]. After stable transduction of Huh-7.5 cells with the VEEV/NS3–5B vector to produce Huh-7.5[VEEV/NS3–5B] cells, the HCV NS3–5B polyprotein was expressed at physiologically relevant levels and properly processed through proteolytic cleavage, as evidenced by the patterns of NS3 and NS5A expression (Fig 1G). NS3–5B expression was stable for many weeks of passage. As a negative control, the green fluorescent protein (GFP) was expressed as an irrelevant protein via the VEEV vector (VEEV/GFP).

We next investigated whether SGR-Gluc(5Bm1) could be *trans*-complemented in Huh-7.5[VEEV/NS3–5B] cells. Indeed, SGR-Gluc(5Bm1) replicated and expressed Gluc in Huh-7.5[VEEV/NS3–5B] cells but not in Huh-7.5[VEEV/GFP] cells, although the kinetics of Gluc expression was slower and less robust than from wild-type SGR-Gluc (Fig 1H **)**. In agreement with the Gluc expression data, SGR-Gluc(5Bm1) RNA levels were maintained in Huh-7.5[VEEV/NS3–5B] cells, albeit at reduced levels compared to SGR-Gluc RNA (Fig 1I). In contrast, SGR-Gluc(5Bm1) RNA levels fell below the limit of detection in Huh-7.5[VEEV/GFP] cells (Fig 1I). These data demonstrate that an HCV mutant containing an inactivating mutation of the NS5B polymerase active site can be *trans*-complemented.

To confirm that SGR-Gluc(5Bm1) replicated in Huh-7.5[VEEV/NS3–5B] cells, transfected cells were treated with 1 nM daclatasvir (DCV), a potent inhibitor of HCV RNA replication complex assembly [90–92]. DCV inhibits JFH-1 replication with an effective concentration (EC_50_) of 71 pM and cytostatic concentrations (CC_50_) of >10 μM [90,93]. Treatment with 1 nM DCV abolished replication of SGR-Gluc(5Bm1) in Huh-7.5[VEEV/NS3–5B] cells (Fig 1J), confirming that the observed *trans*-complementation of SGR-Gluc(5Bm1) requires HCV replicase activity.

We next examined the essential features of NS5B supplied in *trans*. As expected, complementation of SGR-Gluc(5Bm1) required an intact polymerase active site in the recoded NS3–5B polyprotein (S1A Fig). Furthermore, expression of NS5B alone, either by introducing an initiator Met codon (Met-NS5B) or by fusion with ubiquitin to ensure an authentic NS5B N-terminal Ser residue (Ubi-NS5B), failed to *trans*-complement SGR-Gluc(5Bm1) (S1B Fig). Processing of Ubi-NS5B into NS5B was confirmed by western blot (S1C Fig), consistent with efficient cleavage of N-terminal Ubi fusions by cellular ubiquitin C-terminal hydrolases [94,95]. These data indicate that expression of NS5B alone is insufficient to *trans*-complement a defect in NS5B polymerase activity, suggesting that NS5B may need to be expressed as part of the NS3–5B polyprotein.

We also examined the *trans*-complementation of a full-length HCV reporter virus, Jc1/Gluc2A [96], containing the 5Bm1 mutation. As expected, this mutant was unable to replicate in Huh-7.5, Huh-7.5[VEEV/GFP], or Huh-7.5[SGR-Neo] cells, but replicated in Huh-7.5[VEEV/NS3–5B] cells (S1D Fig). However, the dynamic range of *trans*-complementation (i.e., the ratio between the highest and lowest Gluc signals) was lower for Jc1/Gluc2(5Bm1) than for SGR-Gluc(5Bm1), consistent with the higher replication efficiency of SGRs vs. full-length HCV genomes [55,97]. As a result, only low titers (≈2.3 x 10^3^ TCID_50_ units/ml) of infectious virus (as assayed on Huh-7.5[VEEV/NS3–5B] cells) were obtained from the full-length 5Bm1 mutant. Since our focus was to characterize the *cis*- and *trans*- activities of NS genes during RNA replication, SGRs were used in further studies because of their superior dynamic range in replication-dependent Gluc expression.

To determine the long-term stability of NS5B *trans*-complementation, we tested the ability of SGR-Neo(5Bm1) to replicate and transduce Neo-expression in Huh-7.5[VEEV/NS3–5B] cells. As shown in Fig 1K, SGR-Neo(5Bm1) formed G418-resistant colonies in Huh-7.5[VEEV/NS3–5B] cells with an efficiency similar to the wild-type SGR-Neo replicon. Following selection with G418, SGR-Neo(5Bm1) replicon-bearing Huh-7.5[VEEV/NS3–5B] cells could be maintained under selection indefinitely. The 5Bm1 mutation (“GNN”) was maintained stably in G418-selected SGR-Neo(5Bm1) replicon-bearing cells, as seen by direct sequencing of the resulting reverse-transcribed and PCR-amplified amplicon (S1E Fig) and by cloning the PCR product and sequencing twenty independent clones. Although reversion is possible, it was not observed in these experiments. Thus, stable replication of the SGR-Neo(5Bm1) replicon was not due to reversion or recombination with the recoded NS5B gene.

We also tested the *trans*-complementation of an NS5B mutant containing inactivating mutations in the polymerase RNA binding groove, SGR-Gluc(5Bm2), as well as a third mutant, SGR-Gluc(5Bm3), which contained both RNA binding and polymerase active site mutations (Table 1). These mutants were *trans*-complemented with efficiencies similar to SGR-Gluc(5Bm1) (Fig 1L). In contrast to prior results [57,61,83], these data establish that defects in NS5B can be complemented in *trans* by expressing NS3–5B outside the context of an actively replicating SGR.

### NS5B protein expression is required in *cis* for RNA replication

**Fig 2:**
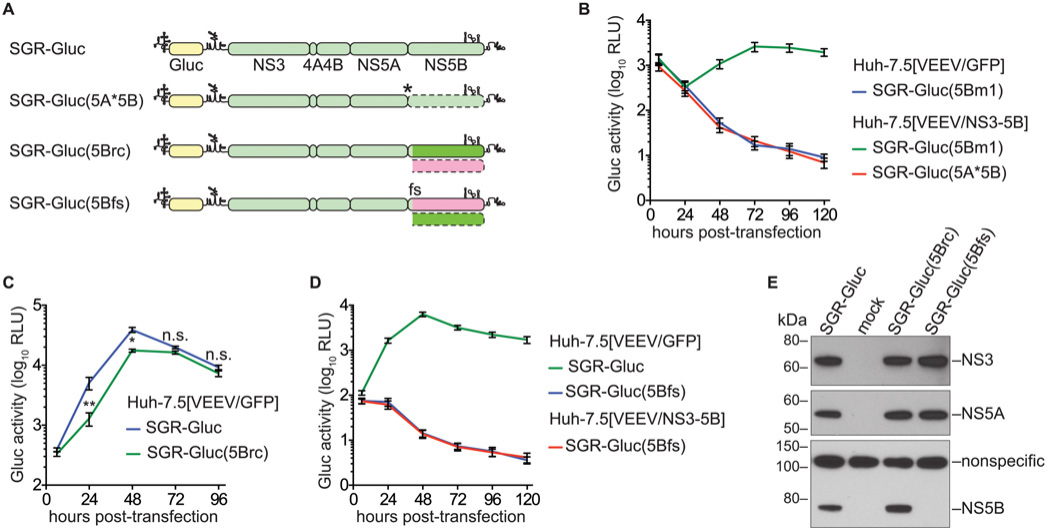
NS5B protein is required in *cis*. (**A**) Constructs used to examine the *cis*-requirement for NS5B expression, as detailed in the text; asterisk, stop codon; dotted lines, untranslated coding regions; fs, -1 frame-shift. For more information, also see Table 1 and S2 Text. (**B**) SGR-Gluc(5A*5B) does not replicate and is not complemented in *trans*. Values represent mean ± SD from transfections done in triplicate and normalized to untransfected controls. This experiment was performed three times with similar results. (**C**) SGR-Gluc(5Brc) replicates efficiently. Values represent mean ± SD from transfections done in triplicate and normalized to untransfected controls; n.s., *p* ≥ 0.05; *, *p* < 0.05; **, *p* < 0.01 by Students *t*-test. This experiment was performed three times with similar results. (**D**) SGR-Gluc(5Bfs) does not replicate and is not replicated in *trans*. Values represent mean ± SD from transfections done in triplicate and normalized to untransfected controls. This experiment was performed three times with similar results. (**E**) The SGR-Gluc(5Brc) and SGR-Gluc(5Bfs) polyproteins are expressed and properly cleaved, as determined by western blot with 4D11 anti-NS3 and 9E10 anti-NS5A monoclonal antibodies. Because these mutants do not replicate, expression was driven by the vaccinia-T7 RNA polymerase system.

We next examined whether the efficiency of NS5B complementation could be improved by preventing expression of the defective NS5B protein. However, the NS5B gene cannot simply be deleted because it contains an RNA structural element, the CRE, required for RNA replication. We therefore inserted a stop codon just downstream of NS5A to create SGR-Gluc(5A*5B) (Table 1 and Fig 2A). This mutant was unable to replicate, but surprisingly, was not complemented in *trans* (Fig 2B). We considered three explanations for these observations. First, the premature stop codon destabilized the SGR-Gluc(5A*5B) RNA. However, SGR-Gluc(5Bm1) and SGR-Gluc(5A*5B) expressed similar levels of residual Gluc (Fig 2B); given that nascent Gluc was collected at each time point (Materials and Methods), these data suggest that non-replicating SGR-Gluc(5Bm1) and SGR-Gluc(5A*5B) RNAs were turned over at similar rates. Second, RNA replication required ribosomal transit through the NS5B coding region, as has been observed for the 2A–3D coding region of poliovirus [11]. Third, the NS5B protein was required in *cis*. To distinguish between these possibilities, we designed a strategy to permit ribosomal translation through the NS5B coding region, without NS5B expression, by using an alternate reading frame. First, we designed the SGR-Gluc(5Brc) mutant containing 22 silent mutations in the NS5B coding region that: 1) removed stop codons from the -1 reading frame; 2) maintained the CRE structure; and 3) maintained a stop at codon 592 (Fig 2A and S2 Text). Thus, this mutant expresses a wild-type NS3–5B polyprotein but uses altered NS5B codon usage to create an untranslated -1 open reading frame. SGR-Gluc(5Brc) autonomously and efficiently replicated, although with slightly delayed kinetics compared to SGR-Gluc (Fig 2C), indicating that these silent mutations in NS5B had only a minor effect on RNA replication.

We then introduced a -1 frame-shift at codon 39 of the SGR-Gluc(5Brc) NS5B gene, to create SGR-Gluc(5Bfs), a mutant predicted to express a 65.3-kDa, 591 amino acid protein, 5Bfs, of unknown structure instead of the 66.7-kDa, 591 amino acid NS5B protein (S2 Text). The SGR-Gluc(5Bfs) mutant did not replicate, nor was it *trans*-complemented (Fig 2D). One possible explanation was that the 5Bfs protein non-specifically interfered with HCV replication. However defects in SGR-Gluc replication were not observed when a 5Bfs transgene was expressed in *trans*, arguing that the 5Bfs-encoded protein did not have a dominant negative effect on replication. We also considered the possibility that the frame-shift could have caused defects in processing of the NS3–5Bfs polyprotein. Because 5Bfs includes the N-terminal 39 amino acids of NS5B, it should allow proper processing of the NS5A/5B junction. Consistent with this, SGR-Gluc(5Bfs) expressed normal levels of NS3 and NS5A but not NS5B (Fig 2E), indicating that proper cleavage of the polyprotein occurred and that normal levels of NS3 and NS5A were expressed. Together, these data show that the recoded NS3–5B transgene was unable to *trans*-complement SGR-Gluc(NS5Bfs), a mutant that: 1) lacks a premature stop codon; 2) allows ribosomes to translate through the NS5B coding region; but 3) does not express NS5B. Thus, two mutants lacking NS5B expression, SGR-Gluc(5A*5B) and SGR-Gluc(5Bfs), were not complemented in *trans*. The simplest interpretation of these data is that RNA replication requires NS5B protein translation in *cis*. Since defects in NS5B RNA binding and polymerase activities can be *trans*-complemented (Fig 1), this *cis*-requirement is therefore independent of the known enzymatic activities of NS5B.

### NS3 serine protease and RNA helicase activities are required in *cis*

**Fig 3:**
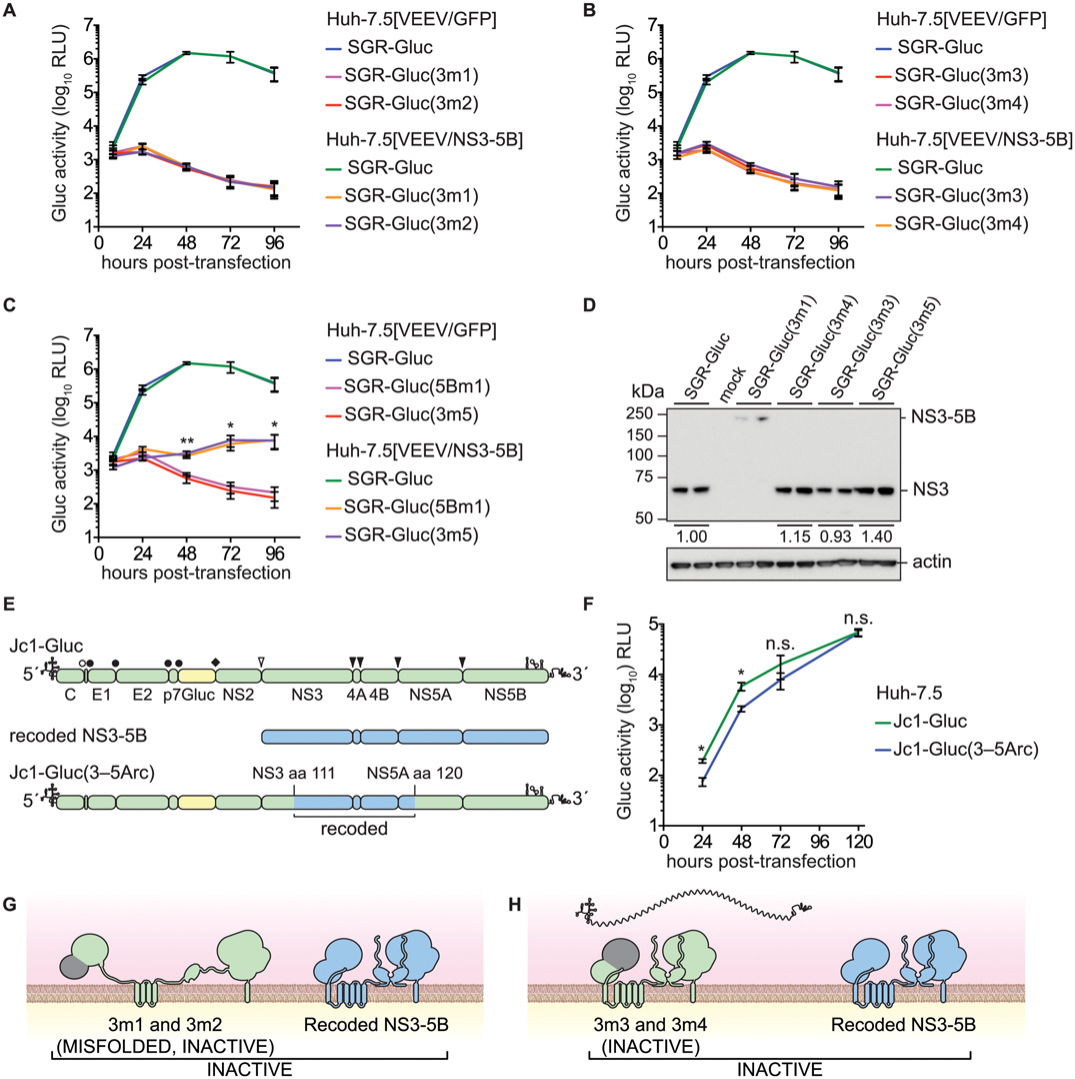
Complementation of NS3 serine protease and RNA helicase active site mutants. (**A**) Serine protease active site mutants SGR-Gluc(3m1) and SGR-Gluc(3m2) do not replicate and are not complemented in *trans*. Values represent mean ± SD from transfections done in triplicate and normalized to untransfected controls. This experiment was performed two times with similar results. (**B**) Helicase RNA binding and NTPase active site mutants SGR-Gluc(3m3) and SGR-Gluc(3m4) do not replicate and are not complemented in *trans*. Values represent mean ± SD from transfections done in triplicate and normalized to untransfected controls. For comparison, the SGR-Gluc samples, which were performed in parallel, are reproduced from panel A. This experiment was performed two times with similar results. (**C**) RNA helicase base stacking mutant SGR-Gluc(3m5) does not replicate but is complemented in *trans* with an efficiency similar to SGR-Gluc(5m1). For comparison, the SGR-Gluc samples, which were performed in parallel, are reproduced from panel A. Values represent mean ± SD from transfections done in triplicate and normalized to untransfected controls; *, *p* < 0.05; **, *p* <0.01 by Student’s *t*-test, comparing matched time points from SGR-Gluc(3m5) in Huh-7.5[VEEV/NS3–5B] vs. Huh-7.5[VEEV/GFP] cells. This experiment was performed two times with similar results. (**D**) Western blot to confirm that the serine protease active site mutant SGR-Gluc(3m1) does not undergo polyprotein cleavage and that the RNA helicase mutants SGR-Gluc(3m2), SGR-Gluc(3m3), SGR-Gluc(3m4), and SGR-Gluc(3m5) do not exhibit defects in NS3 accumulation. NS3 was detected by using anti-NS3 monoclonal antibody 4E11; values represent relative NS3 levels compared to SGR-Gluc. Because these mutants do not replicate, expression was driven by the vaccinia-T7 RNA polymerase system. (**E**) Design of the Jc1-Gluc(3–5Arc) mutant. (**F**) Jc1-Gluc(3–5Arc) replicates efficiently. Values represent mean ± SD from transfections done in triplicate and normalized to untransfected controls; n.s., *p* >0.05, *, *p* ≤ 0.05 by Student’s *t*-test. This experiment was performed two times with similar results (**G**) Model showing that SGR-Gluc(3m1) and SGR-Gluc(3m2) do not undergo proteolytic processing, produce misfolded, inactive polyproteins, and are not complemented in *trans*; grey signifies the nonfunctional serine protease domains; blue signifies the recoded NS3-5B polyprotein. (**H**) Model showing that SGR-Gluc(3m3) and SGR-Gluc(3m4) are not *trans*-complemented and may have a defect in RNA recruitment (compare to Fig 1B); grey signifies the nonfunctional helicase domains; blue signifies the recoded NS3-5B polyprotein.

Given the success in *trans*-complementing NS5B, we examined whether defects in other HCV NS genes could be *trans*-complemented in Huh-7.5[VEEV/NS3–5B] cells. We initially tested mutants containing lethal, inactivating mutations of the NS3 serine protease active site residues (Table 1), SGR-Gluc(3m1) and SGR-Gluc(3m2). Neither of these protease mutants replicated, nor were they complemented in *trans* (Fig 3A). We also examined NS3 RNA helicase domain mutants SGR-Gluc(3m3) and SGR-Gluc(3m4), which contained loss-of-function mutations abrogating RNA binding and NTPase activity, respectively (Table 1). Neither of the helicase domain mutants replicated, nor were they complemented in *trans* (Fig 3B). In comparison, a third helicase domain mutant, SGR-Gluc(3m5) was *trans*-complemented at levels similar to the 5Bm1 mutant (Table 1 and Fig 3C). As detailed in the Discussion, SGR-Gluc(3m5) is a loss-of-function mutant that lacks RNA unwinding activity but retains NS3 helicase RNA binding and NTPase activities [75–77].

Although the data above suggested that HCV replication required the enzymatic activities of NS3 in *cis*, two alternative interpretations are that these specific NS3 mutations: 1) destabilized the inactive NS3 proteins, which could be required in *cis*; or 2) disrupted underlying RNA structures within the NS3 gene that are required in *cis*. Western blot analysis indicated that the 3m3 and 3m5 mutations caused slight (≤2-fold) increases in NS3 accumulation, and that the 3m4 mutation had no effect on NS3 accumulation (Fig 3D). As expected, the 3m1 mutation abrogated serine protease cleavage activity and caused accumulation of an unprocessed NS3–5B polyprotein (Fig 3D). These data show that the helicase mutations did not inhibit NS3 protein accumulation. To examine whether the NS3 mutations disrupted a putative underlying RNA structure required for replication, we replaced a large portion of a full-length HCV genome with the corresponding NS3-5A region from the recoded NS3–5B cassette, creating the Jc1-Gluc(3-5Arc) mutant (Fig 3E and Table 1). This mutant contained 524 silent mutations, including the NS3 RNA binding and NTPase active site codons T269, D290, and E291 that had been mutated in 3m3 and 3m4 (S3 Text). As expected, the recoded NS3 gene also exhibited pronounced differences in predicted RNA secondary structure (S2 Fig). The Jc1-Gluc(3-5Arc) mutant replicated efficiently with only a slight delay in kinetics (Fig 3F); it is therefore unlikely that the 3m3 and 3m4 mutations disrupted an important RNA element in the NS3 gene region. Given that the inactivating NS3 helicase mutations did not inhibit NS3 protein accumulation nor disrupt important RNA secondary structures, we conclude that HCV RNA replication requires the RNA binding and NTPase activities of the NS3 RNA helicase domain in *cis*. While the serine protease activity is required in *cis* because it is needed for polyprotein processing (Fig 3G), the RNA binding and NTPase activities of the helicase domain are likely needed for a post-translational step in replication, such as RNA template recruitment (Fig 3H).

### *trans*-complementation of NS3–5B genes

**Fig 4:**
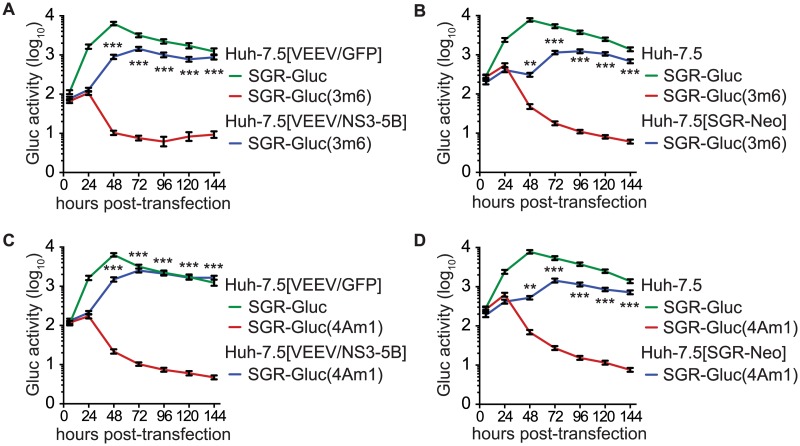
HCV NS3-4A can be complemented in *trans*. (**A**) Efficient replication of SGR-Gluc(3m6) in Huh-7.5[VEEV/NS3–5B] cells but not in Huh-7.5[VEEV/GFP] cells. The replication of SGR-Gluc in Huh-7.5[VEEV/GFP] cells is shown for comparison. (**B**) Replication of SGR-Gluc(3m6) in Huh-7.5[SGR-Neo] cells but not in Huh-7.5 cells. The replication of SGR-Gluc in Huh-7.5 cells is shown for comparison. (**C**) Replication of SGR-Gluc(mut4A) in Huh-7.5[VEEV/NS3–5B] cells but not in Huh-7.5[VEEV/GFP] cells. This experiment was performed in parallel with the experiment shown in (A); the replication of SGR-Gluc in Huh-7.5 cells is reproduced for comparison. (**D**) Replication of SGR-Gluc(4Am1) in Huh-7.5[SGR-Neo] cells but not in Huh-7.5 cells. This experiment was performed in parallel with the experiment shown in (B); the replication of SGR-Gluc in Huh-7.5 cells is reproduced for comparison. All values represent mean ± SD from transfections done in triplicate and normalized to untransfected controls; *, *p* < 0.05; **, *p* < 0.01; ***, *p* <0.001 by Students t-test, comparing matched Gluc activity of SGR-Gluc(3m6) or SGR-Gluc(4Am1) in complementing vs. non-complementing cell lines at each time point. Each experiment was performed twice with similar results.

We also examined the *trans*-complementation of additional NS3-4A mutants. The SGR-Gluc(3m6) mutant, which contains a loss-of-function, three-codon deletion in the NS3 linker region [78], replicated to high levels in Huh-7.5[VEEV/NS3–5B] cells (Fig 4A), indicating that it was *trans*-complemented. This mutant was also complemented in Huh-7.5[SGR-Neo] cells (Fig 4B), indicating that, unlike NS5B, NS3 can be complemented by an actively replicating replicon. Similarly, the SGR-Gluc(4Am1) mutant, which contains a lethal codon substitution in the NS4A acidic domain [65,79], replicated in both Huh-7.5[VEEV/NS3–5B] cells (Fig 4C) and Huh-7.5[SGR-Neo] cells (Fig 4D). Efficient *trans*-complementation was also observed for mutants SGR-Gluc(3m7) and SGR(4Am2), which contained lethal mutations in the NS3 linker and NS4A transmembrane domains, respectively (Table 1 and S2C Fig). Together, these data confirm that NS3-4A can be complemented in *trans*, either by expressing a recoded NS3–5B region or by an actively replicating SGR.

Prior work has shown that NS4B and NS5A can be supplied in *trans* [57–61]. Consistent with these results, SGR-Gluc(4Bm1) and SGR-Gluc(5Am1) (Table 1) had severe replication defects in Huh-7.5[VEEV/GFP] cells but were *trans*-complemented in Huh-7.5[VEEV/NS3–5B] cells (S3A and S3B Fig). Both mutants also were complemented in Huh-7.5[SGR-Neo] cells (S3C and S3D Fig), confirming that defects in NS4B and NS5A can be complemented in *trans* by expressing the wild-type gene either from an active replicon or from a synthetic mRNA encoding NS3–5B.

**Fig 5:**
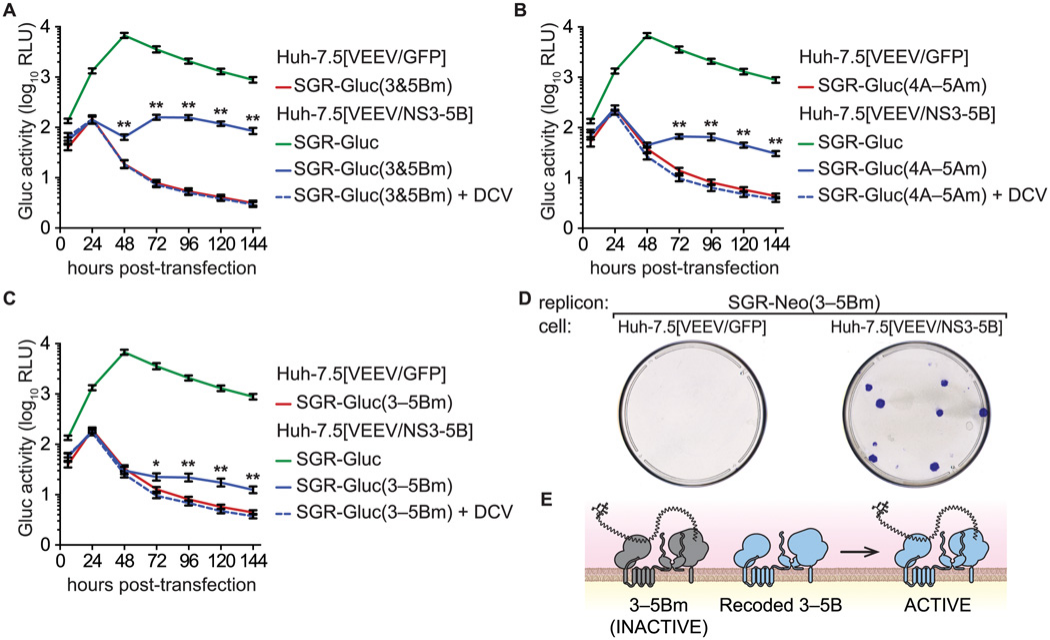
Complementation of NS3–5B in *trans*. (**A**) Complementation of NS3 and NS5B in *trans*. Replication of SGR-Gluc(3&5Bm) and SGR-Gluc was tested in the indicated cell lines. Cultures were treated with DCV (dashed line). For comparison, the replication of SGR-Gluc in Huh-7.5[VEEV/NS3–5B] cells (performed in parallel) is shown. (**B**) Complementation of NS4A, NS4B, and NS5A in *trans*. Replication of SGR-Gluc(4A–5Am) was tested in the indicated cell lines and plotted as in (A). This experiment was performed in parallel with the experiment shown in (A); the replication of SGR-Gluc in Huh-7.5[VEEV/NS3–5B] cells is reproduced for comparison. (**C**) Complementation of NS3-NS5B in *trans*. Replication of SGR-Gluc(3–5Bm) was tested in the indicated cell lines and plotted as in (A). All values represent mean ± SD from transfections done in triplicate and normalized to untransfected controls; *, *p* < 0.05; **, *p* < 0.01; by Student’s t-test, comparing matched time points of each mutant in Huh-7.5[VEEV/NS3–5B] vs. Huh-7.5[VEEV/GFP] cells. Each experiment was performed twice with similar results. (**D**) Stable replication of SGR-Neo(mut3,4A,4B,5A,5B) in Huh-7.5[VEEV/NS3–5B] cells. G418-resistant colonies were stained with crystal violet. (**E**) Model showing that the replication of SGR-Neo(3–5Bm) (grey) is *trans*-complemented by recoded NS3–5B (blue).

We then examined whether multiple defects could be complemented simultaneously by combining two (3m6 and 5Bm1), three (4Am1, 4Bm1, and 5Am1), or five inactivating mutations (3m6, 4Am1, 4Bm1, 5Am1, and 5Bm1) within the SGR-Gluc replicon (Table 1). All of the combined mutants replicated in Huh-7.5[VEEV/NS3–5B] cells but not in Huh-7.5[VEEV/GFP] cells (Fig 5A–5C). The efficiency of complementation inversely correlated with the number of mutations; nevertheless, replication levels were statistically significant and sensitive to DCV treatment, confirming that these signals were due to active HCV replication. None of the combined mutants replicated in Huh-7.5[SGR-Neo] cells (S4A–S4C Fig). To examine the long-term *trans*-complementation of a mutant containing multiple defects, we used G418 selection of a mutant SGR-Neo replicon. As shown in Fig 5D, SGR-Neo(3–5Bm) stably replicated and formed G418-resistant colonies in Huh-7.5[VEEV/NS3–5B] cells but not in Huh-7.5[VEEV/GFP] cells. RT-PCR and sequence analysis of twelve independent cDNA clones confirmed that the 3m6, 4Am1, 4Bm1, 5Am1, and 5Bm1 mutations were all retained in the G418-selected cell populations. These data demonstrate that HCV replicons with combined defects in all five NS genes required for RNA replication (NS3, NS4A, NS4B, NS5A, and NS5B) can be complemented in *trans* (Fig 5E).

### NS3–5B complementation group analysis

**Fig 6:**
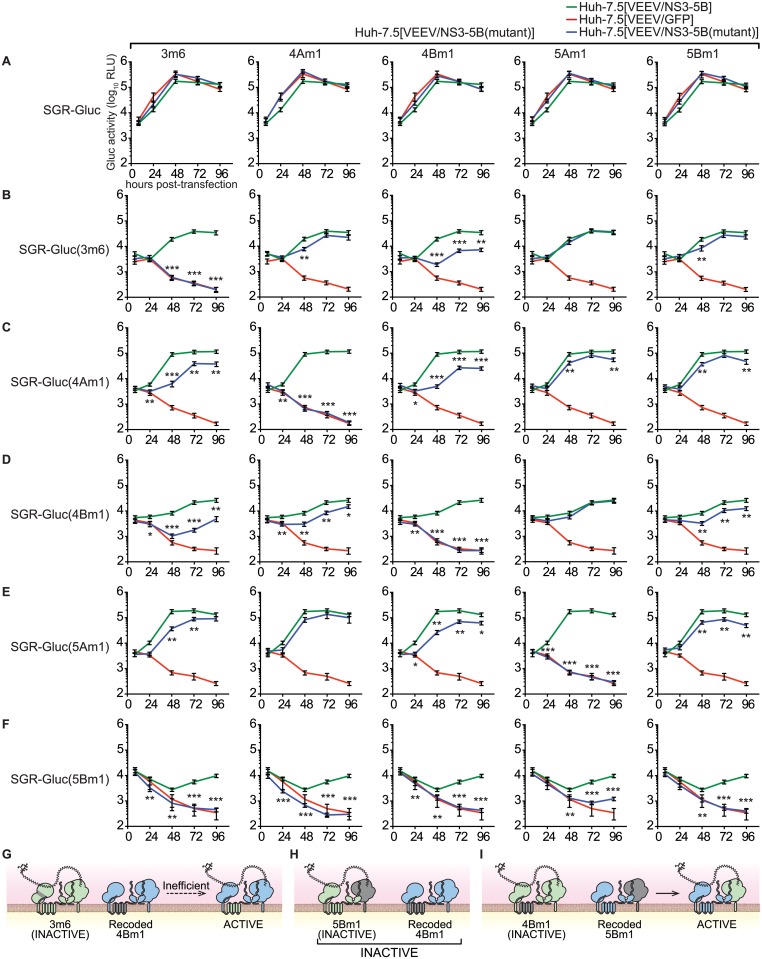
Complementation group analysis. All samples were collected in parallel; therefore, for each mutant, replication in Huh-7.5[VEEV/NS3–5B] and Huh-7.5[VEEV/GFP] cells (green and red lines, respectively) are reproduced for comparison. Values represent mean ± SD from transfections done in triplicate and normalized to untransfected controls; *, *p* < 0.05; **, *p* < 0.01; **, *p* < 0.001 by Student’s t-test, comparing matched time points of each mutant in Huh-7.5[VEEV/NS3–5B(mutant)] cells vs. Huh-7.5[VEEV/NS3–5B] cells. Each experiment was performed twice with similar results. (**A**) SGR-Gluc replication in Huh-7.5[VEEV/NS3–5B] (green), Huh-7.5[VEEV/GFP] (red), or each Huh-7.5[VEEV/NS3–5B] mutant (blue). (**B**) Replication of the SGR-Gluc(3m6) mutant in each cell line. (**C**) Replication of the SGR-Gluc(4Am1) mutant in each cell line. (**D**) Replication of the SGR-Gluc(4Bm1) mutant in each cell line. (**E**) Replication of the SGR-Gluc(5Am1) mutant in each cell line. (**F**) Replication of the SGR-Gluc(5Bm1) mutant in each cell line. (**G**) Model showing that SGR-Gluc(3m6) replication is *trans*-complemented inefficiently in Huh-7.5[VEEV/NS3–5B(4Bm1)] cells. (**H**) Model showing that SGR-Gluc(5Bm1) replication is not complemented in Huh-7.5[VEEV/NS3–5B(4Bm1)] cells. (**I**) Model showing that SGR-Gluc(4Bm1) replication is *trans*-complemented efficiently in Huh-7.5[VEEV/NS3–5B(5Bm1)] cells.

The ability to complement defects in each NS3–5B gene enabled us to examine functional interactions between NS genes through complementation group analysis (Fig 6). To do so, we first created Huh-7.5[VEEV/NS3–5B] cell lines containing the 3m6, 4Am1, 4Bm1, 5Am1, and 5Bm1 mutations within the recoded NS3–5B transgene. Wild-type SGR-Gluc replicated similarly in all cell lines (Fig 6A), indicating that the mutant NS3–5B transgenes did not have dominant negative effects on HCV replication. A panel of SGR-Gluc NS3–5B mutants was then tested for complementation in these cell lines. As expected, each SGR-Gluc mutant failed to replicate in cells expressing the NS3–5B transgene containing a defect in the same gene (Fig 6), thus defining distinct complementation groups. Nonetheless, the efficiency of *trans*-complementation varied among mutant genes, suggesting that functional interactions exist between the corresponding gene products. Notably, the 3m6 mutant replicated less efficiently in Huh-7.5[VEEV/NS3–5B(4Bm1)] cells than in Huh-7.5[VEEV/NS3–5B] cells (Fig 6B); the 4Am1 mutant replicated less efficiently in Huh-7.5[VEEV/NS3–5B(3m6)] and Huh-7.5[VEEV/NS3–5B(4Bm1)] cells than in Huh-7.5[VEEV/NS3–5B] cells (Fig 6C); and the 4Bm1 mutant replicated less efficiently in Huh-7.5[VEEV/NS3–5B(3m6)] cells than in Huh-7.5[VEEV/NS3–5B] cells (Fig 6D). These data indicate that the NS3-4A and NS4B activities were enhanced by co-expression of these genes from the same polyprotein (Fig 6G). However, the most notable effect was that SGR-Gluc(5Bm1) failed to replicate in Huh-7.5[VEEV/NS3–5B(3m6)], Huh-7.5[VEEV/NS3–5B(4Am1)], and Huh-7.5[VEEV/NS3–5B(4Bm1)] cells and was poorly complemented in Huh-7.5[VEEV/NS3–5B(5Am1)] cells (Fig 6F). These data indicate that *trans*-complementing activity of NS5B depends on upstream NS3-5A activities within the same polyprotein (Fig 6H), consistent with our observation that the 5Bm1 mutant was not *trans*-complemented by expression of NS5B alone (S1A Fig). In contrast, the efficient replication of SGR-Gluc(3m6), SGR-Gluc(4Am1), SGR-Gluc(4Bm1), and SGR-Gluc(5Am1) on Huh-7.5[VEEV/NS3–5B(5Bm1)] cells (last column of Fig 6B–6F) indicates that the activity of NS5B in *cis* does not depend on upstream NS3–5A activities (Fig 6I).

### NS5B activity segregates with a DCV-sensitive NS5A activity

**Fig 7:**
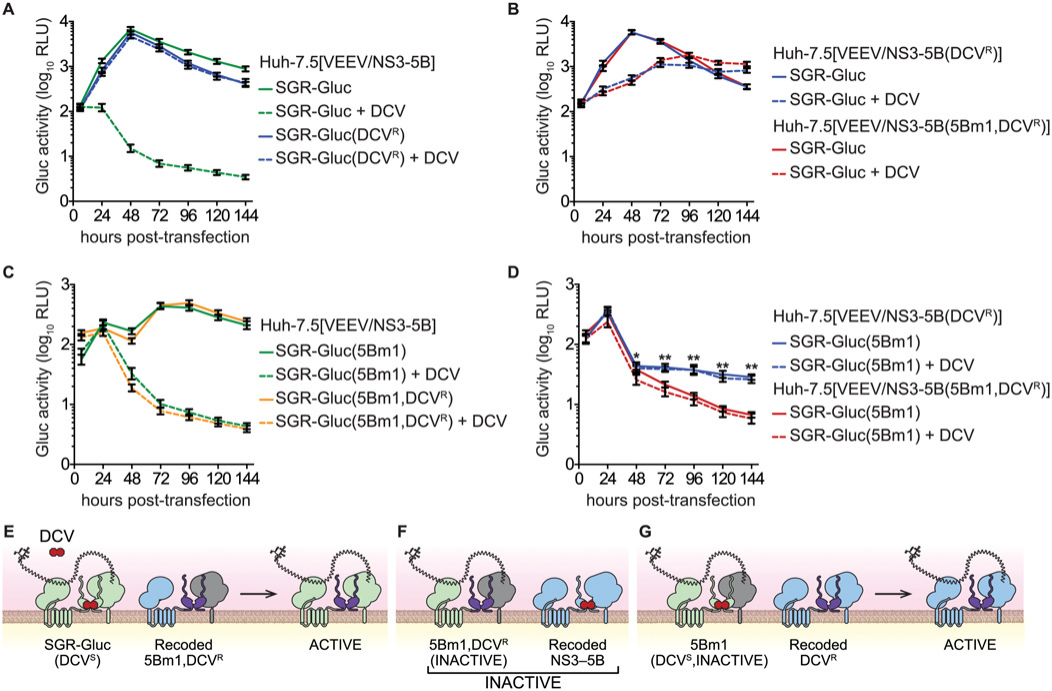
NS5B *trans*-complementation activity segregates with a DCV-sensitive NS5A activity. (**A**) DCV^R^ confers resistance to DCV. Replication of SGR-Gluc and SGR-Gluc(DCV^R^) was tested in Huh-7.5[VEEV/NS3–5B] cells. (**B**) DCV^R^ confers resistance to DCV in *trans*. Replication of SGR-Gluc was tested in Huh-7.5[VEEV/NS3–5B(DCV^R^)] and Huh-7.5[VEEV/NS3–5B(5Bm1,DCV^R^)] cells. (**C**) *trans*-complementation of SGR-Gluc(5Bm1,DCV^R^) is sensitive to DCV. Replication of SGR-Gluc(5Bm1) and SGR-Gluc(5Bm1,DCV^R^) was tested in Huh-7.5[VEEV/NS3–5B] cells. Values represent mean ± SD from transfections performed in triplicate, and normalized to untransfected controls. This experiment was performed twice with similar results. (**D**) Replication of SGR-Gluc was tested in Huh-7.5[VEEV/NS3–5B(DCV^R^)] and Huh-7.5[VEEV/NS3–5B(5Bm1,DCV^R^)] cells. All values represent mean ± SD from independent transfections performed in triplicate and normalized to untransfected controls; *, p<0.05; **, p<0.01 by Student’s *t*-test comparing matched time points to samples treated with 1 nM DCV (dashed lines). All experiments were performed twice with similar results. (**E**) Model showing that SGR-Gluc replication is DCV-insensitive in Huh-7.5[VEEV/NS3–5B(5Bm1,DCV^R^)] cells due to *trans*-complementation of NS5A. (**F**). Model showing that SGR-Gluc(5Bm1,DCV^R^) replication via NS5B *trans*-complementation remains DCV-sensitive in Huh-7.5[VEEV/NS3–5B] cells. (**G**) Model showing that SGR-Gluc(5Bm1) replication is *trans*-complemented and DCV-insensitive in Huh-7.5[VEEV/NS3–5B(DCV^R^)] cells.

*Trans*-complementation can impact the emergence of drug-resistance [2–4]. Given that *trans*-complementation of NS5B is linked to the function of upstream NS3-5A genes, we hypothesized that NS5B activity would segregate with sensitivity to an HCV-specific direct acting antiviral compound targeting an upstream gene product. To explore this hypothesis, we examined the complementation of the lethal 5Bm1 mutation in combination with DCV^R^, a point mutation in NS5A (Table 1) that shifts the EC_50_ of DCV by >7,500-fold and renders HCV resistant to DCV [59]. As expected, replication of the SGR-Gluc(DCV^R^) mutant was resistant to DCV (Fig 7A). When the DCV^R^ mutation was introduced into VEEV/NS3–5B or VEEV/NS3–5B(5Bm1), SGR-Gluc replication was resistant to DCV but showed a kinetic lag (Fig 7B and 7E), again confirming that NS5A can be supplied in *trans*. Surprisingly, *trans*-complementation of the SGR-Gluc(5Bm1,DCV^R^) double mutant was sensitive to DCV (Fig 7C and 7F). However, *trans*-complementation of SGR-Gluc(5Bm1) was resistant to DCV when the DCV^R^ mutation was introduced into VEEV/NS3–5B (Fig 7D and 7G). These data show that NS5A, and in particular, the DCV-sensitive function of NS5A, can function in *trans*, independent of NS5B. However, the *trans*-complementation activity of NS5B segregated with an NS5A activity that was sensitive to DCV, again confirming that NS5B is linked functionally to upstream NS3-5A activities within the same polyprotein.

## Discussion

In this study we define essential *cis*- and *trans*-replication activities for all five HCV replicase genes. Previous studies showed that defects in HCV NS4B and NS5A were complemented in *trans*, whereas defects in NS3 and NS5B were not, leading to the suggestion that NS3-4A and NS5B can only function in *cis* [57–61]. In contrast, we found that specific genetic defects in HCV NS3, NS4A, NS4B, NS5A, and NS5B can be complemented in *trans*, yet other NS3 and NS5B activities are required in *cis*.

**Fig 8:**
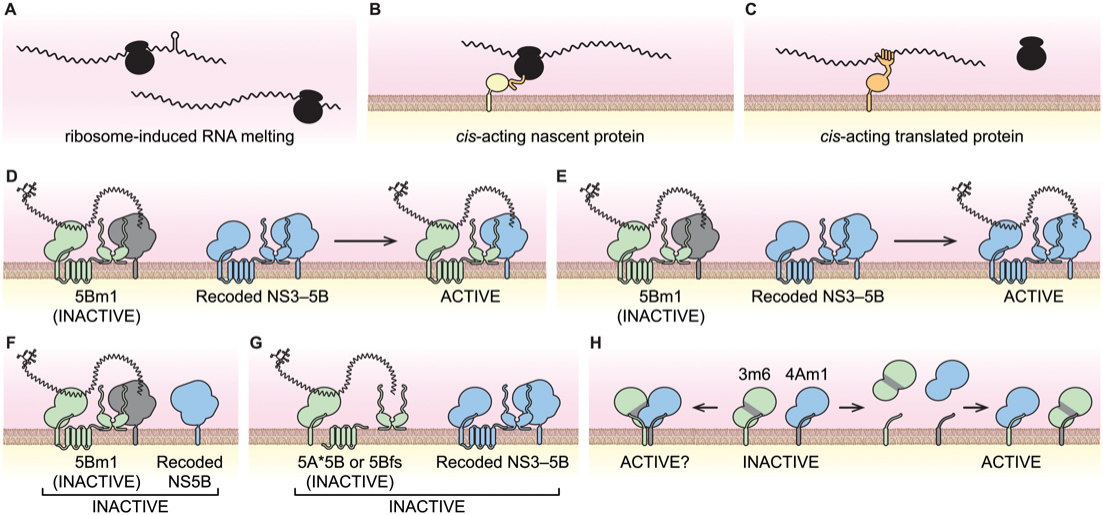
Models of viral *cis*-activities and HCV replicase structure and function. (**A**) Ribosome-induced change in RNA structure. (**B**) Nascent *cis*-acting polypeptide (orange) recruiting the viral RNA-ribosome complex to a site of replication via an interaction partner (yellow). (**C**) A *cis*-acting protein (orange) recruiting the viral RNA to site of replication. (**D**) NS5B *trans*-complementation via protein transfer. (**E**) NS5B *trans*-complementation via RNA transfer. (**F**) NS5B *trans*-complementation requires NS3–5B expression. (**G**) Translation of NS5B is required in *cis*. (**H**) Potential NS3-NS4A interactions of the 3m6 and 4Am1 mutants. NS3-4A can form homodimers where each monomer contributes either NS3 or NS4A (left); alternatively, NS3-NS4A may dissociate and reassociate to form separate active and inactive monomers (right).

How do viral genes and their gene products work in *cis*? In the simplest case, a viral coding region can contain an important RNA sequence or secondary structure, such as the CREs present within the poliovirus 2C gene [15,16] or HCV NS5B gene [17–19]. In other cases, the process of RNA translation is required although the protein product may be irrelevant, such as the ORF encoded by DIs of mouse hepatitis virus [7,20]. As previously noted by Novak and Kirkegaard [11], such *cis*-translational requirements can be explained by ribosome-induced structural changes within the RNA template (Fig 8A), or by recruitment of ribosome-associated factors to the template. Finally, *cis*-acting viral proteins can recruit the RNA from which they were translated into a replication complex. For instance, the nascent N-terminal polypeptide of bovine coronavirus nsp1 targets its RNA template to sites of replication [9]; a similar mechanism has been observed for brome mosaic virus 2a protein [98]. In these cases, the nascent polypeptides are linked physically to the RNA through the actively translating ribosome (Fig 8B). Alternatively, *cis*-acting proteins that have completed synthesis may bind preferentially to their RNA template (Fig 8C) due to spatial limitations, such as physical barriers to protein diffusion, or temporal restrictions, such rapid protein turnover [11]. Similar mechanisms have been put forth to explain *cis*-acting proteins encoded by DNA viruses and other mobile genetic elements [99–104].

### The *cis* and *trans* activities of NS5B

When expressed by an HCV replicon, NS5B exhibited a marked *cis*-preference and was unable to *trans*-complement replicons with defects in NS5B. These data agree with prior studies, which used replicons as the source of NS5B [57,61,83]. However, NS5B could function in *trans* when it was expressed as part of a synthetic NS3–5B polyprotein. We envision two non-exclusive mechanisms by which this could occur within a replication complex: 1) by intermingling of NS proteins and exchange of NS5B (Fig 8D); and/or 2) by transfer of template RNA (Fig 8E). In favor of the RNA transfer model, SGR-Gluc(5Bm1) was not complemented when NS5B was expressed alone (Fig 8F), nor was it complemented by NS3–5B polyproteins containing defects in NS3, NS4A, NS4B, or NS5A (Fig 6H). Thus, *trans*-complementation of NS5B may utilize RNA transfer; at the molecular level, this could reflect a propensity of NS5B to remain associated with the NS proteins with which it was translated. Nevertheless, protein exchange likely occurs during complementation of other viral proteins. For instance NS5A activity segregates promiscuously, independent of other NS protein activities.

While replicons expressing enzymatically inactive NS5B were complemented in *trans*, replicons lacking NS5B expression were not. We excluded nonspecific effects caused by a premature stop codon and a potential requirement for ribosomal transit through the NS5B coding region. These data indicate that NS5B has a *cis*-acting role in replicase assembly that is independent of its RNA binding and polymerase activities. Recent work has shown that the RNA polymerase of poliovirus plays an important, non-enzymatic role in RNA replication by assembling two-dimensional, membrane-bound oligomeric lattices that enhance RNA binding and synthesis through cooperativity [105–107]. Intriguingly, HCV NS5B also forms oligomers that show cooperativity in RNA synthesis rates [108]. Together, these data lend support to a conserved, structural role for viral polymerases during positive-strand RNA virus replicase assembly.

Complementation of HCV NS5B in *trans* only occurred when NS5B was expressed by an intact NS3-5B polyprotein. Analogously, *trans*-complementation of the NS5 polymerase from the related West Nile virus (subtype Kunjin; genus *Flavivirus*) also requires co-expression of upstream NS proteins [86], suggesting this may be common within the *Flaviviridae*. We found that HCV NS5B was functionally linked to NS5A, and more specifically, to the DCV-sensitive function of NS5A translated from the same polyprotein. Fridell and colleagues used SGR *trans*-complementation to define three NS5A complementation groups and showed that the DCV-sensitive function of NS5A works preferentially in *cis* [59,60]. In contrast, we found that the DCV^R^ allele of NS5A can function in *trans* when it was expressed from a synthetic NS3–5B mRNA. Thus, the *cis*-preferences of NS5A and NS5B can be overcome by expressing NS3–5B outside the context of a functional replicon.

Complementation of NS5A also requires expression of an intact NS3-5A polyprotein [57,62] or an active replicon [57–61]. Similar results were observed for NS5A complementation with bovine viral diarrhea virus-1 (BVDV-1), a related member of the *Flaviviridae* [13]. These observations may be related to the influence of upstream proteins on the phosphorylation status and interactome of NS5A [109–112]. Thus, there appears to be multiple layers of *cis*-linkage, whereby NS5B activity is dependent on upstream NS5A activity (this study), which in turn is linked to the activities of NS3-4A and NS4B. This network of linkages could serve as a quality control mechanism to reduce the emergence of DI genomes despite the ability of NS3-5A to function in *trans*. In this regard, deletions in the NS3–5B region have not been observed in naturally occurring HCV deletion mutants [55,113–115].

Given that both NS5A and NS5B are targets of HCV-specific antivirals [23,116], our findings may have implications for the emergence of drug resistance. We predict that NS5A inhibitors like DCV may have synergistic effects when combined with inhibitors of the viral polymerase. Furthermore, the barrier for resistance to drugs targeting NS5A and NS5B may be high because replicase-expressed NS5B does not complement in *trans* and is linked to the daclatasvir-sensitive activity of upstream NS5A; thus resistant alleles would need to arise within the same genome. It is notable that DCV and sofosbuvir combination therapy shows high rates of sustained virological response in HCV-infected patients [117].

### The *cis* and *trans* activities of NS3-4A

Defects in the essential NS3 serine protease, RNA binding, and NTPase active sites were not *trans*-complemented. This phenotype was not unexpected for the serine protease mutants, which express uncleaved, inactive NS3–5B polyproteins (Fig 3G). These data suggest that a protease inhibitor-sensitive virus would not be *trans*-complemented by an inhibitor-resistant virus *in vivo*. Defects in the NS3 RNA binding and NTPase activities also were not complemented in *trans*. We excluded nonspecific effects caused by instability of the mutant NS3 proteins or disruption of underlying *cis*-acting RNA structures within this region of the viral genome. Our data are most consistent with a requirement for the HCV NS3 RNA binding and NTPase activities in *cis*. Similarly, for other *Flaviviridae*, the BVDV-1 NS3 NTPase active site residues are required in *cis* [13,118], whereas a West Nile virus NS3 helicase mutant was *trans*-complemented with very low efficiency [14].

Surprisingly the 3m5 mutant, which contained a lethal W501A mutation of a helicase “bookend” residue, was complemented in *trans*. The NS3 W501 residue has a key role in RNA unwinding. Structurally, W501 stacks against the 3'-most base of RNA and DNA template strands and may keep the template in register for up to three single-nucleotide translocation steps [72,74,76]. *In vitro*, NS3 proteins containing the W501A mutation retain RNA binding and NTPase activities [73,75,119]; such mutants have defects in RNA unwinding but can unwind DNA [75,77]. Thus, the NS3 W501A mutant, SGR/Gluc(3m5), has basal helicase activity but likely has a defect in processivity on RNA templates. While it has been established that HCV helicase activity is required for RNA replication [34,35], the function of the helicase has been unclear, including whether it binds to or unwinds viral RNA during replication. Given that post-translational *cis*-preferences rely on protein-RNA interaction (Fig 8C), our data suggest that the RNA binding and NTPase activities, but not RNA unwinding activity, contribute to RNA template recruitment (Fig 3H). Remarkably, the RNA helicase/NTPase of brome mosaic virus also recruits viral RNA into membrane-bound replication complexes [120]. Thus, RNA template recruitment may be a conserved function of positive-strand RNA virus-encoded helicases.

Defects in the NS3 linker domain and NS4A C-terminal acidic domain also were complemented, both by recoded NS3–5B and by replicons. The NS3 linker domain, which is conserved and essential for RNA replication, coordinates interaction between the protease and helicase domains and may mediate protein-protein interaction via a putative SH3-binding motif [78]. The NS4A acidic domain contributes to helicase activity by promoting RNA-coupled ATP hydrolysis [37] and affects interactions with downstream NS4B and NS5A proteins [65,79]. The helicase activity of recombinant NS3-4A protein containing the 4Am1 mutation (NS4A Y45A) has efficient basal RNA-binding activity, but severely reduced ATP-coupled RNA binding, RNA-stimulated ATPase activity, and RNA unwinding [37]. Thus, the 4Am1 mutant independently confirms that RNA unwinding activity of the NS3-4A helicase can be supplied in *trans*.

Based on the ability to complement representative defects in NS3, NS4A, NS4B, NS5A, and NS5B, we conducted a detailed complementation group analysis for the HCV NS3–5B genes. These experiments revealed functional interactions between NS3-4A and NS4B: 3m6- and 4Am1-mutant replicons were *trans*-complemented less efficiently in cells expressing the 4Bm1-mutant form of NS3–5B, whereas 4Am1- and 4Bm1-mutant replicons were *trans*-complemented less efficiently in cells expressing the 3m6-mutant form of NS3–5B. One interpretation of these data is that NS3-4A and NS4B preferentially interact with each other from the same polyprotein (Fig 6G). Given that the NS3-4A-4B complementation grouping was reciprocal, it was surprising that 3m6- and 4Bm1-mutant replicons replicated well on cells expressing the 4Am1-mutant form of NS3–5B. Furthermore, it was surprising that NS3 and NS4A activities segregated independently given that proteolytic processing of the NS3/NS4A junction is autocatalytic [121] and is predicted to lead to *cis*-preferential interaction between NS3 and NS4A [122]. One possible explanation is that NS3-4A dimerizes, such that one monomer provides NS3 activity while the other provides NS4A activity (Fig 8H). Indeed, we recently demonstrated that the NS4A transmembrane domain mediates homodimerization that is essential for RNA replication [80]. Alternatively, a second explanation might be that NS3-4A heterodimers exchange partners to form a single fully active NS3-4A heterodimer (Fig 8H).

### Complete replication in *trans*

We were able to complement an HCV SGR containing loss-of-function mutations in every replication gene (Fig 5E). It is notable that a few HCV “minigenome” systems have been developed [123–127]. HCV minigenomes are RNAs encoding a reporter gene flanked by the HCV 5' and 3' noncoding regions; because they lack NS protein expression, minigenomes are presumably replicated in *trans* by HCV replicons or NS3–5B transgene expression. Given the *cis*-requirements of NS3 and NS5B described here, it is surprising that minigenomes can be replicated in *trans*. However, minigenome replication levels have not been compared directly to replicon or infectious virus replication levels, nor has their sensitivity to HCV-specific antiviral compounds been demonstrated. A key feature of these systems is that minigenome RNAs are produced continuously through transcription of transfected cDNA; hence they may represent a single round of RNA synthesis rather than *bona fide* RNA replication and amplification. In this regard, NS5B is capable of nonspecific RNA synthesis, in the absence of other HCV NS proteins, when overexpressed in cell culture [128,129]. In contrast, the *trans*-replication system we describe here supports authentic, long-term replication of defective HCV replicons initiated by RNA transfection.

### Conclusions

In summary, we have identified several *cis*- and *trans*-acting activities of the HCV NS3–5B genes that are essential for RNA replication, including a non-enzymatic, *cis*-acting role of NS5B in replicase assembly, and that NS3 RNA binding and NTPase activities are likely involved in template recruitment. NS3-4A and NS4B activities co-segregated in complementation group analysis, whereas NS5B activity was dependent on the function of upstream NS3-5A genes. Together, these studies reveal a network of functional interactions that could serve as a quality control mechanism for the HCV replicase genes and can be exploited by combination therapies targeting multiple NS protein activities.

## Materials and Methods

### cDNA constructs and RNA transcription

SGR-Gluc was previously described as pYSGR-JFH/Gluc [65]. The noncytopathic VEEV vector 5'VEEVrep/L/GFP/Pac [84], a kind gift of Dr. Ilya Frolov (University of Alabama, Birmingham), was modified to replace the GFP insert with a short multi-cloning site. Briefly, oligos YO-0779 and YO-0780 (S1 Table) were annealed and ligated into the XbaI/BsrGI sites of p5'VEEVrep/L/GFP/Pac to yield pVEEV/MCS.

The JFH-1 NS3–5B region was recoded by GeneArt (Regensburg, Germany) to optimize human codon usage, minimize RNA structure, and remove repeat sequences, RNA instability motifs, and AT-rich or GC-rich sequences (S1 Text). The recoded NS3–5B region was subcloned as a 6066-bp PacI/Ecl136II fragment into pVEEV/MCS cut with PacI and PmeI. pJc1-Gluc(3–5Arc) was created by subcloning the SpeI/BstEII fragment of recoded NS3–5B into pJc1-Gluc2A [96]. To make pSGR-Gluc(5Brc), a recoded NS5B–HCV 3' end (S3 Text) was synthesized by Blue Heron Biotech (Bothell, WA) and cloned into pSGR-Gluc as a BsrGI/XbaI fragment. pSGR-Gluc(5Bfs) was constructed by cleaving pSGR-Gluc(NS5Brc) with BsrGI, fill-in with dNTPs and Klenow enzyme, followed by religation. pJc1-Gluc(3–5Arc) was constructed by subcloning a SpeI/BstEII fragment from the recoded NS3–5B region into pJc1/Gluc2A [96].

Mutations were introduced into SGR-Gluc or VEEV/NS3–5B by site-directed mutagenesis with appropriate primer pairs (S1 Table). SGR-Gluc(5Bm1) was described previously as pYSGR-JFH/Gluc(GNN) [65]. SGR-Gluc(4Am1) was constructed by subcloning the NS4A Y45A mutation from Jc1/Gluc2A (NS4A Y45A) [65]. The recoded Met-NS5B gene was created via PCR by using primers YO-0913 and YO-0908. The recoded Ubi-NS5B gene was constructed by overlap extension PCR with primers YO-0905, YO-0906, YO-0907, and YO-0908.

VEEV/NS3-5B is a large (16.6-kb) high copy number plasmid, and cloning efficiency was improved by using high capacity *E*. *coli* strains such as XL10-Gold (Agilent). In contrast, we were unable to obtain a VEEV vector expressing an unrecoded NS3-5B cassette (to test whether the recoding process itself enabled *trans*-complementation) despite numerous cloning attempts with several bacterial host strains. Given that the recoded NS3-5B cassette was successfully subcloned into the VEEV vector several times, we suspect that the authentic JFH-1 NS3-5B sequence adversely impacted stability of the pVEEV plasmid. Nevertheless, the recoded NS3–5B region functioned in *trans* and was used for our studies.

All constructs were confirmed by restriction digestion and/or Sanger sequencing. The sequence of all DNA segments that were amplified *in vitro* were confirmed and subcloned. HCV RNA and VEEV transcripts were produced as previously described [87,130]. RNA transcripts were treated with DNase I (Ambion) and purified with the Qiagen RNeasy kit with on-column DNase digestion [130]. RNA concentrations were checked by spectrophotometry, adjusted to 100 ng/μl, RNA integrity was checked by agarose gel electrophoresis, and 10 μl aliquots were stored at—80°C.

### Cell culture and RNA transfection

Huh-7.5 human hepatoma cells were maintained in complete growth medium (Dulbecco’s modified Eagle medium (DMEM; Invitrogen) containing 10% heat-inactivated fetal calf serum (Omega Scientific) and 0.1 mM non-essential amino acids (Invitrogen)). Cells were transfected with VEEV RNAs by electroporation as described [130]. Two days after electroporation, transfected cells were selected in complete growth medium containing 5 μg/ml puromycin (Invivogen), passaged 3 to 4 times, then frozen. Thawed cells were maintained in puromycin-containing complete growth medium and used within 4 or 5 passages; cells were seeded for experiments in complete growth medium lacking puromycin. Cells were transfected with HCV replicons by using 1 μl RNA and the TransIT mRNA transfection reagent (Mirus) according to the manufacturer’s instructions. DCV was a kind gift of Mingjun Huang (Achillion Pharmaceuticals). For selection of stable replicon-bearing cells, the cells were transfected, as above, in 10 cm dishes. At two days post-transfection, the medium was changed to complete growth medium containing 1 mg/ml G418 (Invitrogen) and selected for 3 weeks with triweekly media changes. Colonies were fixed with methanol and stained with crystal violet [79].

### Luciferase assays

At each time point, media were collected, cells were washed three times with phosphate buffered saline, and fresh media was added. The collected media were clarified by centrifugation (5 min at 21,130 x *g*), placed into fresh tubes, mixed with 1/4 volume 5x Lysis buffer (Promega or NEB), and stored frozen (-80°C). Samples were assayed for Gluc activity on a Berthold Centro LB 960 plate reader [96] or a Berthold Centro XS^3^ LB 960.

### Protein analysis

Cell lysates were prepared and equal amounts of protein were electrophoresed by SDS-PAGE, transferred to polyvinylidene difluoride membranes and immunoblotted with NS3-specific monoclonal antibodies 9G2 (Virogen) or 4E11 (this study), NS5A-specific monoclonal antibody 9E10 [130], NS5B-specific monoclonal antibody 5D1 (BioFront), or ß-actin-specific monoclonal antibody AC-15 (Sigma) antibodies. We noticed that the NS5B-specific antibody 5D1 had large lot-to-lot variability in NS5B reactivity and non-specific background. Protein masses were estimated based on the migration of the SuperSignal Enhanced Molecular Weight protein ladder (Pierce, Rockford IL). To analyze polyprotein expression of defective replicons, the vaccinia-T7 RNA polymerase expression system was used [131]. Briefly, Huh-7.5 cells were seeded at 5 x 10^5^/well into six-well plates the day before use. Cells were infected for 1 h at a multiplicity of infection (MOI) of 10 with vTF7-3 and transfected with pSGR-Gluc derivatives by using Mirus LT1 reagent according to the manufacturer’s recommendations. Lysates were collected at 24 h post-transfection. Relative NS3 expression (Fig 3D) was quantitated by using the gel analyzer densitometry feature of NIH ImageJ [132].

### NS3 antibody production

Recombinant HCV NS3 protein was expressed and purified from *E*. *coli* as described [133]. BALB/c mice were primed and boosted at 3-week intervals with 25 μg recombinant NS3 protein complexed with adjuvant (RIBI Immunochemical). Approximately 1 month after the last boost, sera were harvested and tested for immunoreactivity against HCV-infected Huh-7.5 cells. Mice with high titers (specific signal at >1/1,000 dilution) were boosted intravenously with 15 μg purified NS3 protein in PBS. Splenocytes were harvested 3 d later and fused to P3X63Ag8.653 myeloma cells to generate hybridomas according to published procedures [134]. A total of 8 hybridomas were derived after repeated subcloning, and monoclonal antibodies were purified by standard protein A/protein G chromatography. Monoclonal antibody 4D11 was found to react with NS3 protein by western blot of HCV-infected cells vs. uninfected control cells. Other monoclonal antibodies developed in these fusions will be described elsewhere.

### RNA analysis

RNA was harvested by using Trizol reagent (Invitrogen). For sequence analysis, HCV RNA was amplified from the selected cell populations by RT-PCR and amplicons were directly submitted for Sanger sequencing; PCR products were also cloned into pCR2.1TOPO (Invitrogen) and multiple independent cDNA clones were sequenced. HCV RNA was quantitated by qRT-PCR as previously described [96] and normalized to total RNA concentration. Briefly, 2 μl of each RNA were assayed in 20 μl reactions containing 200 nM of an HCV IRES-specific hydrolysis probe (6FAM-5'-TAT GAG TGT CGT GCA GCC TC-3'-MGBNFQ), 400 nM forward (5'-CTT CAC GCA GAA AGC GTC TA-3'), 400 nM reverse (5'-CAA GCA CCC TAT CAG GCA GT-3') primers, and the LightCycler RNA Amplification Kit (Roche). Reactions were analyzed in a Roche LightCycler 480 by using a 45 minute RT step at 56°C, a 5 minute denaturation step at 94°C, followed by 40 cycles of denaturation (10 seconds at 95°C), annealing (20 seconds at 60°C), and extension (3 seconds at 72°C). Quantitation curves were constructed by analyzing HCV RNA standards in parallel [96].

### Statistical analysis

All transfection experiments were performed at least twice, with each experiment containing at least three replicate cell cultures that were independently plated, transfected, sampled, and analyzed. Mean relative light unit (RLU) values were calculated ± standard deviations (SD) based on replicate data and plotted against matched control samples collected in parallel. Because many samples could be processed in parallel, the same positive and negative controls are presented across multiple samples within large experiments, as explicitly noted in the Figure legends. Because the background sensitivity of luciferase assays was affected by the lysis buffer and luminometer hardware, data were normalized to untransfected cells by dividing the mean value of each sample by the mean value from matched, mock-transfected cells collected in parallel. To calculate SD values for the normalized data, the propagations of error were accounted for as described [135]. Statistical significance was calculated in GraphPad Prism by using two-tailed, paired Student’s *t*-tests of the normalized, replicate data for each time point.

## Supporting Information

S1 Fig NS5B *trans*-complementation. (**A**) SGR-Gluc(5Bm1) does not replicate in Huh-7.5[VEE/NS3-5B(5Bm1)] cells, indicating that the NS5B active site is required in *trans*. (**B**) SGR-Gluc(5Bm1) is not complemented by expression of NS5B alone via VEE/Met-NS5B or VEE/Ubi-NS5B. (**C**) Western blot confirms that Ubi-NS5B is cleaved, presumably by a ubiquitin C-terminal hydrolase, to produce full-length NS5B. (**D**) Complementation of full-length HCV reporter virus Jc1/Gluc2A(5Bm1) in Huh-7.5[VEE/NS3-5B] cells. All values represent mean ± SD from transfections performed in triplicate and normalized to untransfected controls; experiments were repeated three times with similar results. (**E**) Sanger sequencing of *trans*-complemented SGR-Neo(5Bm1) after three weeks of G418 selection and two weeks of additional passage. Total RNA was extracted from G418-resistant cells with Trizol reagent, HCV-specific cDNA was synthesized, the NS5B gene region was amplified by RT-PCR, and the PCR product was directly sequenced. For comparison, a parallel sequence of VEE/NS3-5B DNA is shown.
(PDF)

S2 Fig Supplementary data for NS3-4A complementation. (**A**) Secondary structure prediction of the SGR-Gluc NS3 gene was calculated by mfold and represented here in a circle plot. The NS3 gene is represented in a clockwise direction starting at 6 o’clock, with key residues and residue numbers indicated around the circle. Arcs within the circle indicate predicted base pairs and are colored by pnum values, as indicated by the color scale. (**B**) Secondary structure prediction of the SGR-Gluc (3–5Arc) NS3 gene, as in (**A**). (**C**) SGR-Gluc(3m7) and SGR-Gluc(4Am2) replicate in Huh-7.5[VEEV/NS3-5B] cells but not Huh-7.5[VEEV/GFP] cells. Values represent mean ± SD from transfections done in triplicate and normalized to untransfected controls.
(PDF)

S3 Fig Complementation of NS4B and NS5A. (**A** and **C**) The replication of SGR-Gluc(4Bm1) was tested in the indicated cell lines in parallel with the experiment shown in Fig 4A and 4B. The replication of SGR-Gluc was reproduced from Fig 4 for comparison. (**B** and **D**) The replication of SGR-Gluc(5A1) was tested in the indicated cell lines, as above. Values represent mean ± SD from transfections performed in triplicate and normalized to untransfected controls. All experiments were repeated three times with similar results.
(PDF)

S4 Fig The combined mutants are not trans-complemented by SGR-Neo. (**A**) The replication of SGR-Neo(3&5Bm) was tested in Huh-7.5 or Huh-7.5[SGR-Neo] cells. (**B**) The replication of SGR-Neo(4A–5Am) was tested in Huh-7.5 or Huh-7.5[SGR-Neo] cells. (**C**) The replication of SGR-Neo(3–5Bm) was tested in Huh-7.5 or Huh-7.5[SGR-Neo] cells. Data were plotted as in Fig 1B. The replication of SGR-Gluc in Huh-7.5 cells, performed in parallel, is shown for comparison. Values represent mean ± SD from transfections performed in triplicate and normalized to untransfected controls. All experiments were repeated three times with similar results.
(PDF)

S1 Table Primers used in these studies. (DOCX)

S1 Text Sequence of recoded NS3-5B. (DOCX)

S2 Text Sequence of NS5Brc. The NS5B gene begins at position 6013 in SGR-Gluc. The region downstream of the BsrGI site was recoded with silent mutations (lower case bp) to remove stop codons from the -1 reading frame. SGR-Gluc(NS5Bfs) was constructed by cutting SGR-Gluc(NS5Brc) BsrGI, refilling to form blunt ends, and religation.
(DOCX)

S3 Text Sequence of NS3rc. The wild-type JFH-1 NS3 gene (WT) was aligned to the NS3rc gene (RC), which contains recoded NS3 sequence downstream of SpeI. Mismatches are indicated by hash marks. The T269, D290, D291, and W501 residues are highlighted for emphasis.
(DOCX)
