# Supplementary figures and images for: Hepatitis C Virus RNA Replication Depends on Specific Cis- and Trans-Acting Activities of Viral Nonstructural Proteins
Source: PLoS Pathog. 2015 Apr 13;11(4):e1004817. doi: 10.1371/journal.ppat.1004817 (PMC4395149; doi:10.1371/journal.ppat.1004817)

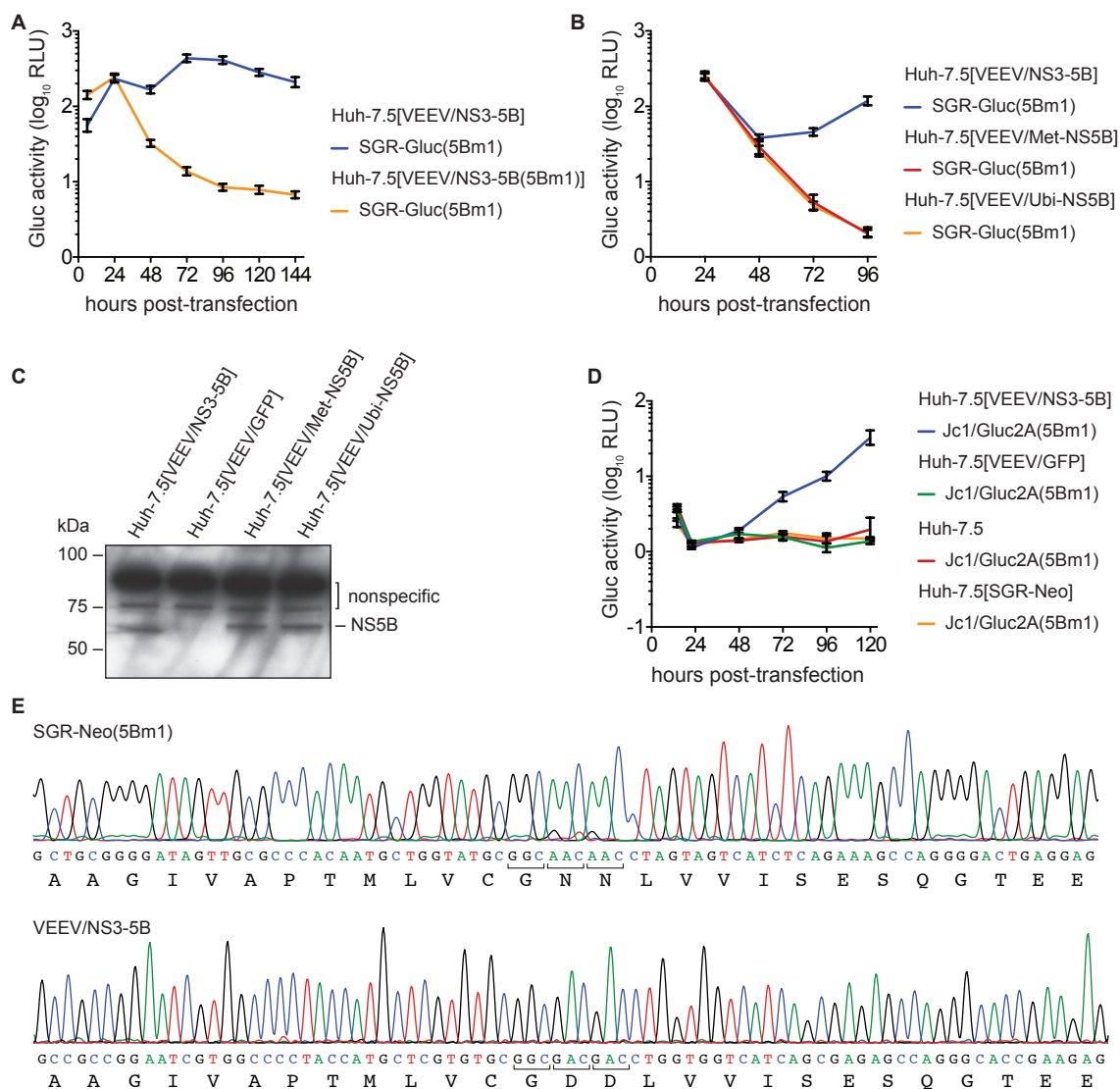

Supplement: S1 Fig — (A) SGR-Gluc(5Bm1) does not replicate in Huh-7.5[VEE/NS3-5B(5Bm1)] cells, indicating that the NS5B active site is required in trans. (B) SGR-Gluc(5Bm1) is not complemented by expression of NS5B alone via VEE/Met-NS5B or VEE/Ubi-NS5B. (C) Western blot confirms that Ubi-NS5B is cleaved, presumably by a ubiquitin C-terminal hydrolase, to produce full-length NS5B. (D) Complementation of full-length HCV reporter virus Jc1/Gluc2A(5Bm1) in Huh-7.5[VEE/NS3-5B] cells. All values represent mean ± SD from transfections performed in triplicate and normalized to untransfected controls; experiments were repeated three times with similar results. (E) Sanger sequencing of trans-complemented SGR-Neo(5Bm1) after three weeks of G418 selection and two weeks of additional passage. Total RNA was extracted from G418-resistant cells with Trizol reagent, HCV-specific cDNA was synthesized, the NS5B gene region was amplified by RT-PCR, and the PCR product was directly sequenced. For comparison, a parallel sequence of VEE/NS3-5B DNA is shown. (PDF) [file ppat.1004817.s001.pdf]

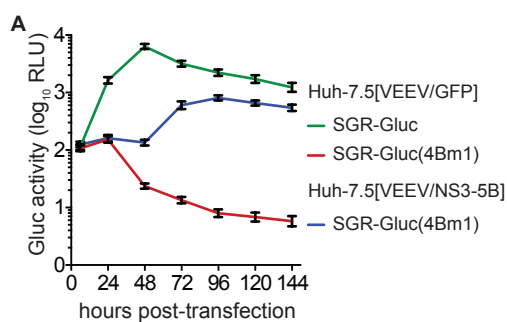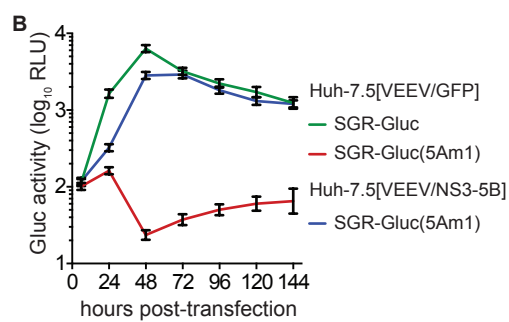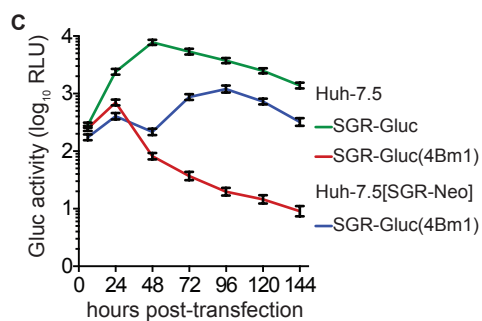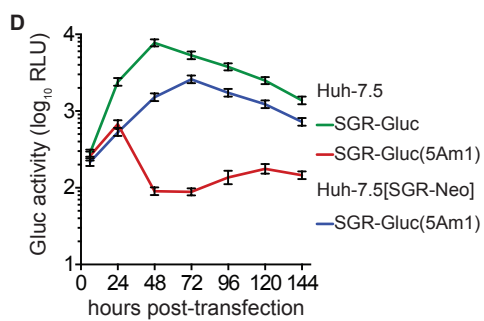

Supplement: S3 Fig — (A and C) The replication of SGR-Gluc(4Bm1) was tested in the indicated cell lines in parallel with the experiment shown in Fig 4A and 4B. The replication of SGR-Gluc was reproduced from Fig 4 for comparison. (B and D) The replication of SGR-Gluc(5A1) was tested in the indicated cell lines, as above. Values represent mean ± SD from transfections performed in triplicate and normalized to untransfected controls. All experiments were repeated three times with similar results. (PDF) [file ppat.1004817.s003.pdf]

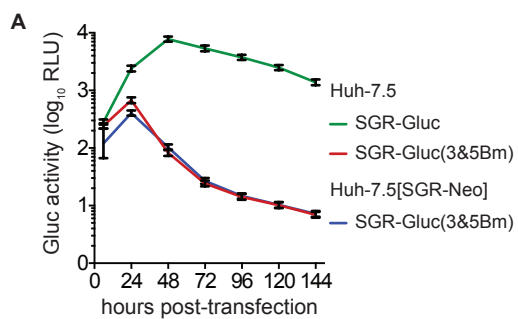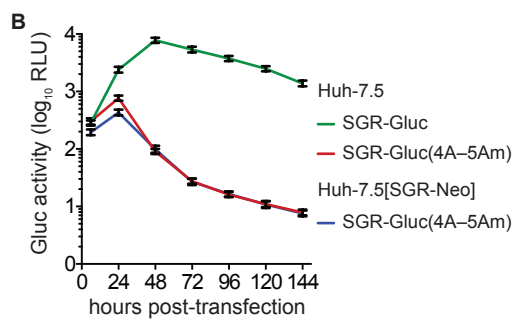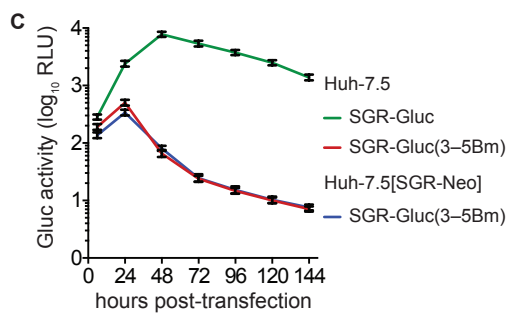

Supplement: S4 Fig — (A) The replication of SGR-Neo(3&5Bm) was tested in Huh-7.5 or Huh-7.5[SGR-Neo] cells. (B) The replication of SGR-Neo(4A–5Am) was tested in Huh-7.5 or Huh-7.5[SGR-Neo] cells. (C) The replication of SGR-Neo(3–5Bm) was tested in Huh-7.5 or Huh-7.5[SGR-Neo] cells. Data were plotted as in Fig 1B. The replication of SGR-Gluc in Huh-7.5 cells, performed in parallel, is shown for comparison. Values represent mean ± SD from transfections performed in triplicate and normalized to untransfected controls. All experiments were repeated three times with similar results. (PDF) [file ppat.1004817.s004.pdf]
