# Supplementary material for: Hepatitis C Virus RNA Replication Depends on Specific Cis- and Trans-Acting Activities of Viral Nonstructural Proteins
Source: PLoS Pathog. 2015 Apr 13;11(4):e1004817. doi: 10.1371/journal.ppat.1004817 (PMC4395149; doi:10.1371/journal.ppat.1004817)
Supplement: S2 Fig — (A) Secondary structure prediction of the SGR-Gluc NS3 gene was calculated by mfold and represented here in a circle plot. The NS3 gene is represented in a clockwise direction starting at 6 o’clock, with key residues and residue numbers indicated around the circle. Arcs within the circle indicate predicted base pairs and are colored by pnum values, as indicated by the color scale. (B) Secondary structure prediction of the SGR-Gluc (3–5Arc) NS3 gene, as in (A). (C) SGR-Gluc(3m7) and SGR-Gluc(4Am2) replicate in Huh-7.5[VEEV/NS3-5B] cells but not Huh-7.5[VEEV/GFP] cells. Values represent mean ± SD from transfections done in triplicate and normalized to untransfected controls. (PDF) [file ppat.1004817.s002.pdf]

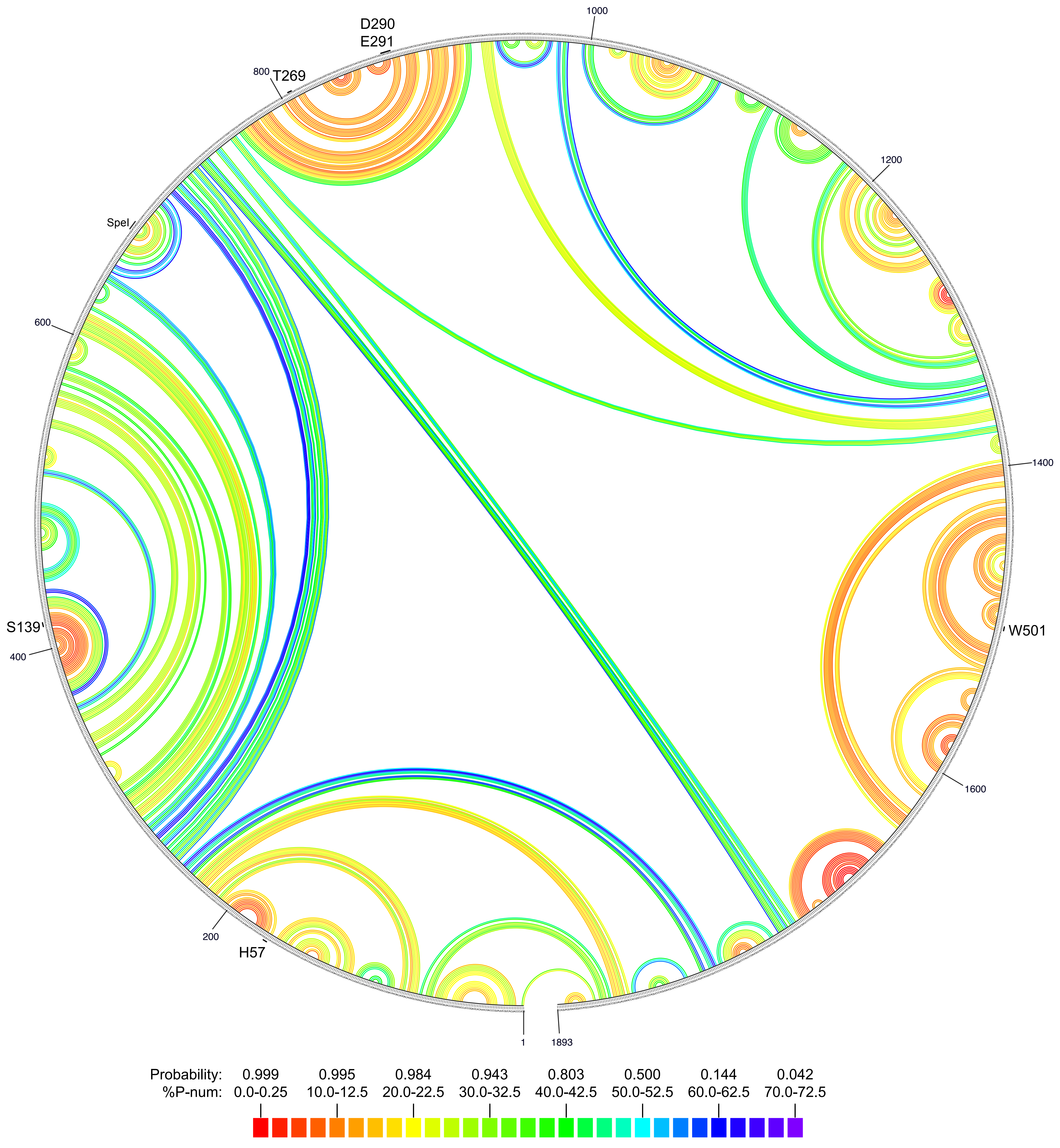

WT NS3 gene
ΔG = -738.17 [Initially -804.70]

Kazakov, et al.
Figure S2A

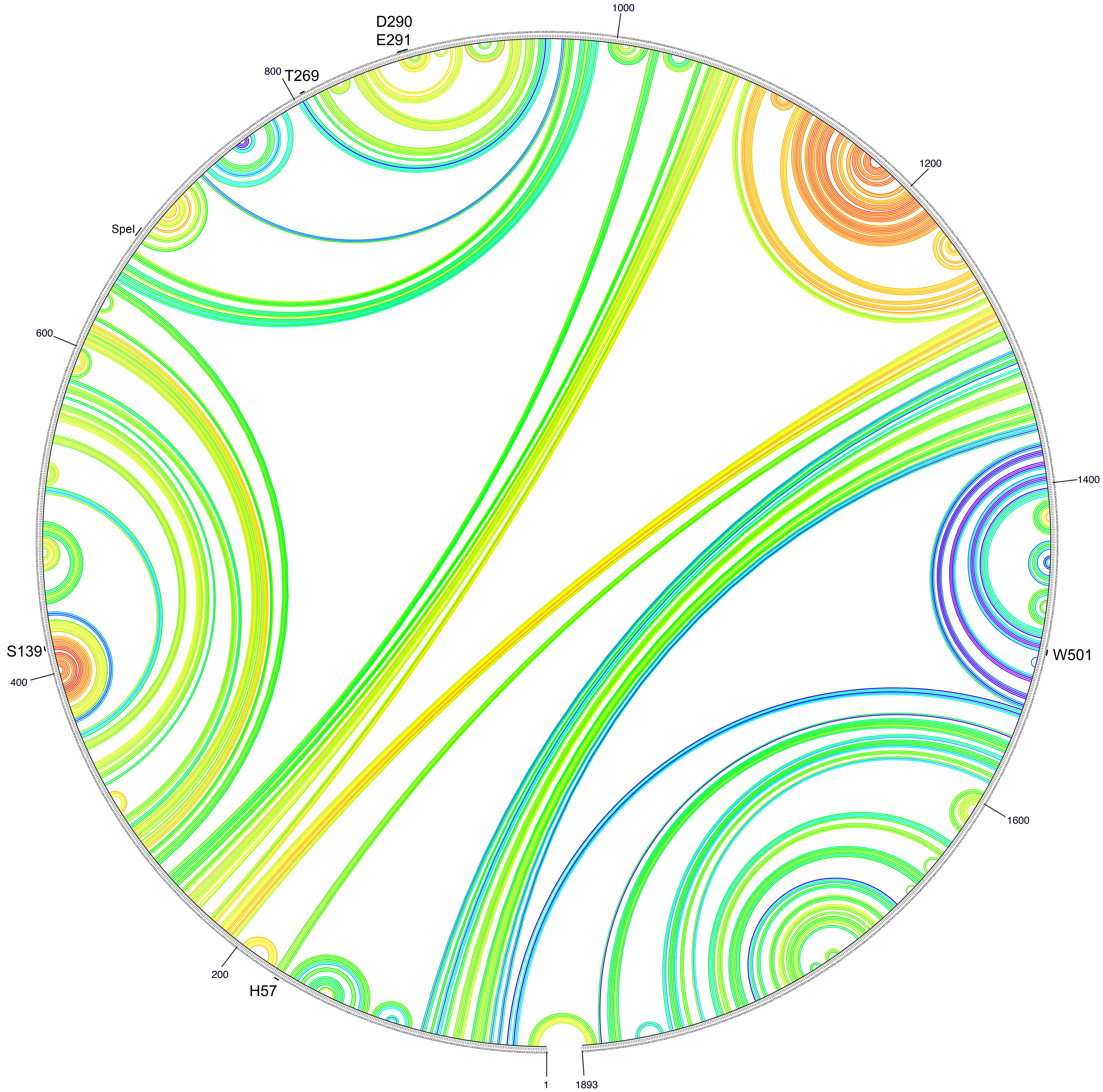

Recoded NS3 gene
ΔG = -729.88 [Initially -805.30]

Kazakov, et al.
S2B Figure

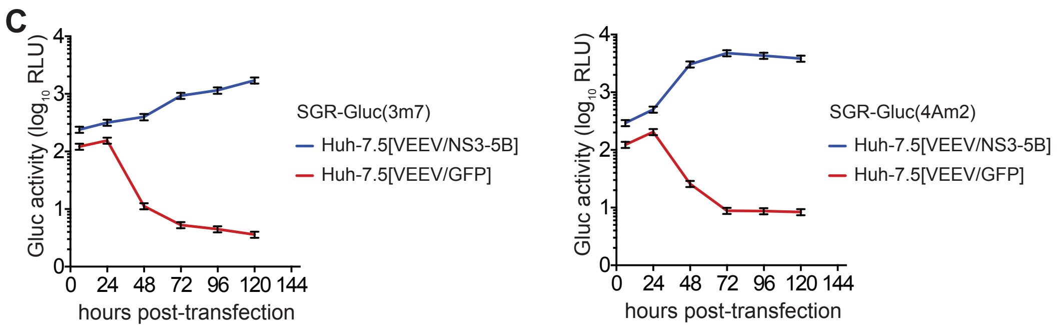

Kazakov, et al., S2C Figure
