# Supplementary material for: Hepatitis C Virus RNA Replication Depends on Specific Cis- and Trans-Acting Activities of Viral Nonstructural Proteins
Source: PLoS Pathog. 2015 Apr 13;11(4):e1004817. doi: 10.1371/journal.ppat.1004817 (PMC4395149; doi:10.1371/journal.ppat.1004817)
Supplement: S1 Table — (DOCX) [file ppat.1004817.s005.docx]

**S1 Table. Primers used in these studies.**

| Primer | Sequence | Use | F/R^*^ |
| --- | --- | --- | --- |
| YO-0779 | 5´-CTA GAT TAA TTA AGG CGC GCC GTT TAA ACT-3´ | VEEV/MCS | F |
| YO-0780 | 5´-GTA CAG TTT AAA CGG CGC GCC TTA ATT AAT-3´ | VEEV/MCS | R |
| YO-0893 | 5´-CCT ACC ATG CTC GTG TGC GGC AAC AAC CTG GTG GTC ATC AGC GAG AGC CAG-3´ | Recoded 5Bm1 | F |
| YO-0894 | 5´-CTG GCT CTC GCT GAT GAC CAC CAG GTT GTT GCC GCA CAC GAG CAT GGT AGG-3´ | Recoded 5Bm1 | R |
| YO-1245 | 5´-TTA CCC TGA CCT CGG CGT CGC GGT CTG CGA GGC AAT GGC CCT CTA TGA CAT TA-3´ | SGR 5Bm2 | F |
| YO-1246 | 5´-TAA TGT CAT AGA GGG CCA TTG CCT CGC AGA CCG CGA CGC CGA GGT CAG GGT AA-3´ | SGR 5Bm2 | R |
| YO-0823 | 5´-GAT ACC ACC GTG TGC TGC TCC ATG TCA TAC TCC TGG ACC-3´ | SGR 5A*5B | F |
| YO-0824 | 5´-GGT CCA GGA GTA TGA CAT GGA GCA GCA CAC GGT GGT ATC-3´ | SGR 5A*5B | R |
| YO-0913 | 5´-TTA ATT GAG GAT CCC GCC ACC ATG AGC ATG AGC TAC AGC TGG AC-3´ | Met-NS5B | F |
| YO-0908 | 5´-GTT TAA ACT TAT CAT CTG GCA GGC AGC AGA AAC AGC-3´ | Met-NS5B | R |
| YO-0905 | 5´-TTA ATT AAC GAG GAT CCC GCC ACC ATG CAG ATC TTC GTG AAG AC-3´ | Ubi-NS5B | F |
| YO-0906 | 5´-GTC CAG CTG TAG CTC ATG CTT CCA CCG CGG AGA CGC AGC ACC-3´ | Ubi-NS5B | R |
| YO-0907 | 5´-GGT GCT GCG TCT CCG CGG TGG AAG CAT GAG CTA CAG CTG GAC-3´ | Ubi-NS5B | F |
| YO-1157 | 5´-GGG TTT TGT GGA CTG TTT ACG CCG GAG CTG GCA ACA AGA CTC-3´ | SGR 3m1 | F |
| YO-1158 | 5´-GAG TCT TGT TGC CAG CTC CGG CGT AAA CAG TCC ACA AAA CCC-3´ | SGR 3m1 | R |
| YO-0911 | 5´-TTT CGA CCT TGA AGG GGT CCG CCG GGG GGC CGG TGC TCT GCC CTA-3´ | SGR 3m2 | F |
| YO-0912 | 5´-TAG GGC AGA GCA CCG GCC CCC CGG CGG ACC CCT TCA AGG TCG AAA-3´ | SGR 3m2 | R |
| YO-2011 | 5´-GGG AGG CCA TCA CGT ACT CCG CCT ATG GCA AAT TTC TCG-3´ | SGR 3m3 | F |
| YO-2012 | 5´-CGA GAA ATT TGC CAT AGG CGG AGT ACG TGA TGG CCT CCC-3´ | SGR 3m3 | R |
| YO-2013 | 5´-TAT GAC ATC ATC ATA TGC GCC GCC TGC CAC GCT GTG GAT-3´ | SGR 3m4 | F |
| YO-2014 | 5´-ATC CAC AGC GTG GCA GGC GGC GCA TAT GAT GAT GTC ATA-3´ | SGR 3m4 | R |
| YO-2015 | 5´-ACG ACG CAG GGG CTG CGG CCT ACG ATC TCA CAC CAG CGG-3´ | SGR 3m5 | F |
| YO-2016 | 5´-CCG CTG GTG TGA GAT CGT AGG CCG CAG CCC CTG CGT CGT-3´ | SGR 3m5 | R |
| AK63F | 5´-ACA AGG TCT CCC ACT AAC AGC ACG CCA CCG-3´ | SGR 3m6 | F |
| AK64F | 5´-CGG TGG CGT GCT GTT AGT GGG AGA TGT-3´ | SGR 3m6 | R |
| YO-1566 | 5´-GGT CAC CAG AAG CCC CAC CAA CAG CAC CCC TCC TGC CGT GCC TCA-3´ | Recoded 3m6 | F |
| YO-1567 | 5´-CGG CAG GAG GGG TGC TGT TGG TGG GGC TTC TGG TGA CCA-3´ | Recoded 3m6 | R |
| YO-1001 | 5´-CCT ACT CAC TAC GTG ACG GCC TCG GAT GCG TCG CAG CGT G-3´ | SGR 4Bm1 | F |
| YO-1002 | 5´-CAC GCT GCG ACG CAT CCG AGG CCG TCA CGT AGT GAG TAG G-3´ | SGR 4Bm1 | R |
| YO-1009 | 5´-CCC ACC CAC TAC GTG ACC GGC AGC GAC GCC AGC CAG AGA G-3´ | Recoded 4Bm1 | F |
| YO-1010 | 5´-CTC TCT GGC TGG CGT CGC TGG CGG TCA CGT AGT GGG TGG G-3´ | Recoded 4Bm1 | R |
| YO-1005 | 5´-GAG GCG AGC TCC TCA GTG ATC CAG CTA TCA GCA CCG TCG C-3´ | SGR 5Am1 | F |
| YO-1006 | 5´-GCG ACG GTG CTG ATA GCT GGA TCA CTG AGG AGC TCG CCT C-3´ | SGR 5Am1 | R |
| YO-1572 | 5´-AGG CCA GCA GCT CCG TGA TCC AGC TGT CCG CCC CTA-3´ | Recoded 5Am1 | F |
| YO-1573 | 5´-TAG GGG CGG ACA GCT GGA TCA CGG AGC TGC TGG CCT-3´ | Recoded 5Am1 | R |
| YO-0899 | 5´-CTG ACC TCT AAA TTG AGC CCT AAG CTG CCC GGC CTC CCC T -3´ | SGR DCV^R^ | F |
| YO-0900 | 5´-GGG AGG CCG GGC AGC TTA GGG CTC AAT TTA GAG GTC AG-3´ | SGR DCV^R^ | R |
| YO-0901 | 5´-GGC TGA CCA GCA AGC TGT CTC CCA AGC TGC CCG GCC TGC C-3´ | Recoded DCV^R^ | F |
| YO-0902 | 5´-GGC AGG CCG GGC AGC TTG GGA GAC AGC TTG CTG GTC AGC C-3´ | Recoded DCV^R^ | R |

*F, forward; R, reverse.
