# Supplementary material for: Hepatitis C Virus RNA Replication Depends on Specific Cis- and Trans-Acting Activities of Viral Nonstructural Proteins
Source: PLoS Pathog. 2015 Apr 13;11(4):e1004817. doi: 10.1371/journal.ppat.1004817 (PMC4395149; doi:10.1371/journal.ppat.1004817)
Supplement: S1 Text — (DOCX) [file ppat.1004817.s006.docx]

**S1 Text. Sequence of recoded NS3-5B.**

LOCUS recoded_NS3_5B 6072 bp ds-DNA LINEAR 07-SEP-2013

DEFINITION Recoded HCV genotype 2a strain JFH-1 NS3-5B genes

ACCESSION

VERSION

SOURCE Synthetic

ORGANISM Hepatitis C virus

Viruses; ssRNA positive-strand viruses, no DNA stage; Flaviviridae;

Hepacivirus.

COMMENT Codon optimization performed by GeneArt to optimize human codon

COMMENT usage, minimize RNA structure, and remove repeat sequences, RNA

COMMENT instability motifs, and AT-rich or GC-rich sequences.

FEATURES Location/Qualifiers

CDS 39..1931

/label=recoded JFH NS3

CDS 1932..2093

/label=recoded JFH NS4A

CDS 2094..2876

/label=recoded JFH NS4B

CDS 2877..4274

/label=recoded JFH NS5A

CDS 4275..6048

/label=recoded JFH NS5B

ORIGIN

1 TTAATTAAGA GGTACCTCTA GAGGATCCCG CCACCATGGC CCCTATCACC GCCTACGCCC

61 AGCAGACAAG AGGACTGCTG GGAGCCATCG TGGTGTCCAT GACCGGCAGA GACAGAACAG

121 AGCAGGCCGG CGAGGTGCAG ATCCTGAGCA CCGTGTCCCA GAGCTTTCTG GGCACCACCA

181 TCAGCGGCGT GCTGTGGACC GTGTACCATG GCGCTGGCAA CAAGACACTG GCCGGCCTGA

241 GAGGCCCTGT GACCCAGATG TACAGCAGCG CCGAGGGCGA TCTCGTGGGC TGGCCTTCAC

301 CTCCTGGCAC CAAGAGCCTG GAACCCTGCA AGTGTGGCGC CGTGGACCTG TACCTGGTCA

361 CCCGGAACGC CGATGTGATC CCCGCCAGAA GAAGAGGCGA CAAGAGAGGC GCCCTGCTGT

421 CCCCCAGACC CATCAGCACC CTGAAGGGCA GCAGCGGAGG CCCAGTGCTG TGTCCTAGAG

481 GCCACGTCGT GGGCCTGTTT AGAGCCGCCG TGTGCAGCAG AGGCGTGGCC AAGAGCATCG

541 ACTTCATCCC CGTGGAGACA CTGGACGTGG TCACCAGAAG CCCCACCTTC AGCGACAACA

601 GCACCCCTCC TGCCGTGCCT CAGACCTACC AGGTCGGCTA TCTGCACGCA CCTACCGGCA

661 GCGGCAAGTC TACCAAGGTG CCCGTGGCCT ATGCCGCCCA GGGCTACAAG GTACTAGTGC

721 TGAACCCCAG CGTGGCCGCC ACACTGGGCT TTGGCGCCTA CCTGAGCAAG GCCCACGGCA

781 TCAACCCCAA CATCCGGACC GGCGTGAGAA CCGTGATGAC CGGCGAGGCC ATCACCTACA

841 GCACCTACGG CAAGTTTCTG GCCGATGGCG GCTGTGCCAG CGGCGCCTAC GACATCATCA

901 TCTGCGACGA GTGCCACGCC GTCGACGCCA CAAGCATCCT GGGCATCGGC ACCGTGCTGG

961 ACCAGGCCGA AACCGCTGGC GTGAGACTGA CCGTGCTGGC CACCGCTACC CCACCTGGCA

1021 GCGTGACCAC CCCCCACCCC GACATCGAGG AAGTGGGCCT GGGCAGAGAG GGCGAGATCC

1081 CCTTCTACGG CAGAGCCATC CCCCTGAGCT GCATCAAGGG CGGCAGACAC CTGATCTTTT

1141 GCCACAGCAA GAAGAAGTGC GACGAGCTGG CCGCTGCCCT GAGAGGCATG GGCCTGAACG

1201 CCGTGGCCTA CTACAGAGGC CTGGACGTGT CCATCATCCC TGCCCAGGGC GACGTGGTCG

1261 TCGTCGCCAC CGATGCCCTG ATGACAGGCT ACACCGGCGA CTTCGACAGC GTGATCGACT

1321 GCAACGTGGC CGTGACCCAG GCCGTGGACT TCAGCCTGGA CCCTACCTTC ACCATCACCA

1381 CCCAGACCGT GCCACAGGAC GCCGTGAGCA GAAGCCAGCG GAGAGGCAGA ACAGGCAGAG

1441 GCCGGCAGGG CACCTACAGA TACGTGTCCA CCGGCGAGAG GGCCAGCGGC ATGTTCGACT

1501 CCGTGGTGCT GTGCGAGTGC TACGATGCCG GCGCTGCTTG GTACGATCTG ACCCCTGCCG

1561 AGACAACCGT GCGGCTGCGG GCCTACTTCA ACACCCCTGG CCTGCCCGTG TGTCAGGACC

1621 ACCTGGAATT CTGGGAGGCC GTGTTCACCG GCCTGACCCA CATCGACGCC CACTTTCTGA

1681 GCCAGACCAA GCAGGCAGGG GAGAACTTCG CCTACCTGGT CGCCTATCAG GCAACAGTGT

1741 GCGCCAGAGC CAAAGCCCCT CCCCCTAGCT GGGACGCCAT GTGGAAGTGC CTGGCCAGAC

1801 TGAAGCCTAC CCTGGCCGGA CCTACCCCCC TGCTGTACAG ACTGGGCCCC ATCACCAACG

1861 AAGTGACCCT GACCCACCCC GGCACCAAGT ATATCGCCAC CTGTATGCAG GCCGACCTGG

1921 AAGTGATGAC CTCTACCTGG GTGCTGGCTG GCGGAGTGCT GGCCGCCGTC GCCGCTTATT

1981 GTCTGGCCAC CGGCTGCGTG AGCATCATCG GCCGGCTGCA CGTGAACCAG AGAGTGGTGG

2041 TCGCCCCCGA CAAAGAGGTG CTGTACGAGG CCTTCGACGA GATGGAAGAG TGCGCCAGCA

2101 GAGCAGCCCT GATCGAGGAA GGCCAGCGGA TCGCCGAGAT GCTGAAGTCC AAGATCCAGG

2161 GACTGCTCCA GCAGGCTAGC AAGCAGGCCC AGGACATCCA GCCTGCCATG CAGGCCTCTT

2221 GGCCCAAGGT GGAGCAGTTC TGGGCCAGAC ACATGTGGAA CTTCATCAGC GGCATCCAGT

2281 ACCTGGCTGG CCTGAGCACC CTGCCTGGCA ATCCAGCCGT GGCCAGCATG ATGGCCTTCT

2341 CTGCCGCCCT CACCAGCCCT CTGTCTACCT CCACCACCAT CCTCCTGAAC ATCATGGGCG

2401 GCTGGCTGGC TTCTCAGATC GCCCCTCCTG CTGGAGCCAC AGGCTTCGTG GTGTCTGGCC

2461 TCGTGGGAGC CGCTGTGGGC AGCATCGGCC TGGGCAAGGT GCTGGTCGAT ATCCTGGCCG

2521 GCTACGGCGC TGGAATTAGC GGAGCCCTGG TGGCTTTCAA GATCATGTCT GGCGAGAAGC

2581 CCAGCATGGA AGATGTGATC AATCTGCTCC CCGGCATCCT GAGCCCTGGC GCTCTGGTCG

2641 TGGGCGTGAT CTGTGCCGCC ATCCTGAGAA GGCACGTGGG ACCTGGTGAA GGCGCTGTGC

2701 AGTGGATGAA CCGGCTGATC GCCTTCGCTA GCAGGGGCAA TCACGTGGCC CCCACCCACT

2761 ACGTGACCGA GAGCGACGCC AGCCAGAGAG TGACTCAGCT GCTGGGCTCT CTGACCATCA

2821 CCAGCCTGCT GCGGAGACTG CACAACTGGA TCACCGAGGA CTGCCCCATC CCTTGTAGCG

2881 GCAGCTGGCT GAGGGACGTG TGGGACTGGG TCTGCACCAT CCTGACCGAC TTCAAGAACT

2941 GGCTGACCAG CAAGCTGTTC CCCAAGCTGC CCGGCCTGCC CTTCATCAGC TGCCAGAAGG

3001 GCTATAAGGG CGTCTGGGCC GGCACAGGCA TCATGACCAC CAGATGCCCC TGCGGCGCTA

3061 ACATCTCCGG CAATGTGCGG CTCGGCAGCA TGAGAATCAC CGGCCCCAAG ACCTGTATGA

3121 ACACCTGGCA GGGCACATTC CCCATCAACT GCTACACCGA GGGCCAGTGC GCCCCTAAGC

3181 CCCCCACAAA CTACAAGACC GCCATCTGGC GGGTGGCCGC CTCTGAGTAC GCCGAGGTCA

3241 CCCAGCACGG CAGCTACAGC TACGTGACAG GCCTGACCAC CGACAACCTG AAGATCCCTT

3301 GCCAGCTGCC CAGCCCCGAG TTCTTCAGCT GGGTGGACGG CGTGCAGATC CACAGATTCG

3361 CCCCCACCCC CAAGCCTTTC TTCCGGGACG AGGTGTCCTT CTGCGTGGGC CTGAATAGCT

3421 ACGCCGTGGG CAGCCAGCTG CCTTGCGAGC CTGAGCCCGA TGCCGACGTG CTGCGGAGCA

3481 TGCTGACCGA CCCCCCTCAC ATCACAGCCG AGACAGCCGC CAGAAGGCTG GCCAGAGGCA

3541 GCCCTCCTAG CGAGGCCAGC AGCTCCGTGT CTCAGCTGTC CGCCCCTAGC CTGAGAGCCA

3601 CCTGTACCAC CCACAGCAAC ACCTACGACG TGGACATGGT GGACGCCAAT CTGCTGATGG

3661 AAGGCGGCGT GGCCCAGACA GAGCCCGAGA GCAGAGTGCC CGTGCTGGAT TTCCTGGAAC

3721 CCATGGCCGA GGAAGAGAGC GACCTGGAAC CTAGCATCCC CAGCGAGTGC ATGCTGCCTA

3781 GAAGCGGCTT CCCTAGAGCC CTGCCTGCCT GGGCTAGGCC TGACTACAAC CCCCCCCTGG

3841 TGGAGTCTTG GCGGAGGCCC GACTACCAGC CTCCTACCGT GGCTGGATGC GCCCTGCCCC

3901 CTCCTAAGAA GGCCCCTACC CCCCCTCCTC GGCGGAGAAG AACAGTGGGC CTGAGCGAGT

3961 CCACAATCAG CGAGGCCCTG CAGCAGCTGG CCATCAAGAC CTTCGGCCAG CCTCCAAGCA

4021 GCGGAGATGC CGGCAGCTCT ACAGGCGCTG GCGCCGCTGA ATCTGGCGGC CCTACCTCTC

4081 CTGGCGAGCC TGCCCCTAGC GAGACAGGCA GCGCCAGCAG CATGCCTCCT CTGGAAGGCG

4141 AGCCCGGCGA CCCTGACCTG GAAAGCGACC AGGTGGAGCT GCAGCCACCT CCTCAGGGGG

4201 GAGGTGTGGC TCCTGGCAGC GGCAGCGGCT CTTGGAGCAC CTGTAGCGAA GAGGACGACA

4261 CCACCGTGTG TTGCAGCATG AGCTACAGCT GGACAGGCGC CCTGATCACC CCTTGCAGCC

4321 CTGAGGAAGA GAAGCTGCCC ATCAACCCCC TGAGCAACAG CCTGCTGAGA TACCACAACA

4381 AGGTGTACTG CACCACCTCC AAGAGCGCCT CCCAGCGGGC CAAGAAAGTG ACCTTCGACC

4441 GGACCCAGGT GCTGGACGCC CACTACGACA GCGTGCTGAA GGACATCAAA CTGGCCGCCA

4501 GCAAGGTGTC AGCCCGGCTG CTGACACTGG AAGAGGCCTG CCAGCTGACC CCTCCTCACT

4561 CTGCCAGAAG CAAGTACGGC TTCGGAGCCA AAGAAGTGCG GAGCCTGAGC GGCAGAGCCG

4621 TGAACCACAT CAAGAGCGTG TGGAAGGATC TGCTGGAAGA TCCCCAGACC CCCATCCCTA

4681 CCACCATCAT GGCCAAGAAC GAGGTGTTCT GCGTGGACCC TGCCAAGGGC GGAAAGAAGC

4741 CCGCCAGACT GATCGTGTAC CCCGACCTGG GCGTGAGAGT GTGCGAGAAG ATGGCCCTGT

4801 ACGACATCAC CCAGAAGCTG CCTCAGGCCG TCATGGGCGC CAGCTACGGC TTCCAGTACA

4861 GCCCTGCCCA GAGAGTGGAG TATCTGCTGA AGGCCTGGGC CGAGAAGAAA GACCCCATGG

4921 GCTTCAGCTA TGACACCCGG TGCTTCGACA GCACAGTGAC CGAGCGGGAC ATCAGAACCG

4981 AGGAAAGCAT CTACCAGGCC TGCAGCCTCC CTGAAGAAGC CAGAACCGCC ATCCACAGCC

5041 TGACCGAGAG ACTGTACGTG GGCGGACCCA TGTTCAACAG CAAGGGCCAG ACCTGTGGCT

5101 ACAGACGGTG CAGAGCCTCT GGCGTGCTGA CCACCTCCAT GGGCAATACC ATCACCTGTT

5161 ACGTGAAGGC CCTGGCCGCC TGTAAAGCCG CCGGAATCGT GGCCCCTACC ATGCTCGTGT

5221 GCGGCGACGA CCTGGTGGTC ATCAGCGAGA GCCAGGGCAC CGAAGAGGAT GAGCGGAACC

5281 TGAGAGCCTT CACCGAGGCC ATGACCAGAT ACAGCGCCCC TCCCGGCGAT CCTCCCAGAC

5341 CCGAGTACGA TCTGGAACTG ATCACCAGCT GCAGCAGCAA CGTGTCTGTG GCACTGGGAC

5401 CCAGGGGCAG AAGGCGGTAC TACCTGACCA GGGACCCCAC AACCCCTCTC GCCAGAGCCG

5461 CCTGGGAAAC AGTGCGGCAC AGCCCCATCA ATAGCTGGCT GGGCAACATC ATCCAGTACG

5521 CCCCCACCAT CTGGGTCCGG ATGGTGCTGA TGACCCACTT CTTCAGCATC CTGATGGTGC

5581 AGGACACCCT GGACCAGAAC CTGAACTTCG AGATGTACGG CTCCGTGTAC AGCGTGAACC

5641 CCCTGGATCT GCCCGCCATC ATCGAGAGAC TGCACGGCCT GGACGCCTTC AGCATGCACA

5701 CCTACAGCCA CCACGAGCTG ACCAGAGTGG CCTCTGCCCT GAGAAAACTG GGAGCCCCAC

5761 CCCTGAGAGT GTGGAAGTCC AGAGCCAGAG CCGTGCGGGC CAGCCTGATT AGCAGAGGCG

5821 GCAAGGCCGC TGTGTGCGGC AGATACCTGT TCAACTGGGC CGTGAAAACC AAGCTGAAGC

5881 TGACCCCCCT CCCTGAGGCG CGCCTGCTCG ACCTGAGCAG CTGGTTTACC GTGGGCGCTG

5941 GCGGAGGCGA TATCTTCCAC AGCGTGTCCC GCGCCAGACC TAGAAGCCTG CTGTTCGGCC

6001 TGCTGCTGCT GTTTGTGGGC GTGGGGCTGT TTCTGCTGCC TGCCAGATGA TAAAGATCTA

6061 AGCTTGAGCT CA

//
