# Supplementary material for: Hepatitis C Virus RNA Replication Depends on Specific Cis- and Trans-Acting Activities of Viral Nonstructural Proteins
Source: PLoS Pathog. 2015 Apr 13;11(4):e1004817. doi: 10.1371/journal.ppat.1004817 (PMC4395149; doi:10.1371/journal.ppat.1004817)
Supplement: S2 Text — The NS5B gene begins at position 6013 in SGR-Gluc. The region downstream of the BsrGI site was recoded with silent mutations (lower case bp) to remove stop codons from the -1 reading frame. SGR-Gluc(NS5Bfs) was constructed by cutting SGR-Gluc(NS5Brc) BsrGI, refilling to form blunt ends, and religation. (DOCX) [file ppat.1004817.s007.docx]

**S2 Text. Sequence of NS5Brc.** The NS5B gene begins at position 6013 in SGR-Gluc. The region downstream of the BsrGI site was recoded with silent mutations (lower case bp) to remove stop codons from the -1 reading frame. SGR-Gluc(NS5Bfs) was constructed by cutting SGR-Gluc(NS5Brc) BsrGI, refilling to form blunt ends, and religation.

6020 6030 6040 6050 6060 6070 6080 6090 6100 6110

* * * * * * * * * * * * * * * * * * * *

6013 TCCATGTCATACTCCTGGACCGGGGCTCTAATAACTCCCTGTAGCCCCGAAGAGGAAAAGTTGCCAATCAACCCTTTGAGTAACTCGCTGTTGCGATACC 6112

S M S Y S W T G A L I T P C S P E E E K L P I N P L S N S L L R Y H

6127 BsrGI

6120 |6130 6140 6150 6160 6170 6180 6190 6200 6210

* * * | * * * * * * * * * * * * * * * * *

6113 ATAACAAGGTGTACTGTACAACATCAAAGAGCGCCTCACAGAGGGCcAAAAAGGTAACTTTcGACAGGACGCAAGTGCTCGACGCCCATTAcGACTCAGT 6212

N K V Y C T T S K S A S Q R A K K V T F D R T Q V L D A H Y D S V

L Y N I K E R L T E G Q K G N F R Q D A S A R R P L R L S

6220 6230 6240 6250 6260 6270 6280 6290 6300 6310

* * * * * * * * * * * * * * * * * * * *

6213 CTTAAAGGACATCAAGCTAGCGGCTTCCAAGGTCAGCGCAAGGCTCCTCACCTTGGAGGAGGCGTGCCAGTTGACTCCACCCCATTCTGCAAGATCCAAG 6312

L K D I K L A A S K V S A R L L T L E E A C Q L T P P H S A R S K

L K G H Q A S G F Q G Q R K A P H L G G G V P V D S T P F C K I Q V

6320 6330 6340 6350 6360 6370 6380 6390 6400 6410

* * * * * * * * * * * * * * * * * * * *

6313 TATGGATTCGGGGCCAAGGAGGTCCGCAGCTTGTCCGGGAGGGCCGTgAACCACATCAAGTCCGTGTGGAAGGACCTCCTGGAAGACCCACAAACACCAA 6412

Y G F G A K E V R S L S G R A V N H I K S V W K D L L E D P Q T P I

W I R G Q G G P Q L V R E G R E P H Q V R V E G P P G R P T N T N

6420 6430 6440 6450 6460 6470 6480 6490 6500 6510

* * * * * * * * * * * * * * * * * * * *

6413 TTCCCACAACCATCATGGCCAAAAAcGAGGTGTTCTGCGTGGACCCCGCCAAGGGGGGcAAGAAACCAGCTCGCCTCATCGTTTACCCcGACCTCGGCGT 6512

P T T I M A K N E V F C V D P A K G G K K P A R L I V Y P D L G V

S H N H H G Q K R G V L R G P R Q G G Q E T S S P H R L P R P R R

6520 6530 6540 6550 6560 6570 6580 6590 6600 6610

* * * * * * * * * * * * * * * * * * * *

6513 CCGGGTCTGCGAGAAAATGGCCCTCTAcGACATTACACAAAAGCTTCCTCAGGCGGTAATGGGAGCTTCCTATGGCTTCCAGTACTCCCCTGCCCAACGG 6612

R V C E K M A L Y D I T Q K L P Q A V M G A S Y G F Q Y S P A Q R

P G L R E N G P L R H Y T K A S S G G N G S F L W L P V L P C P T G

6620 6630 6640 6650 6660 6670 6680 6690 6700 6710

* * * * * * * * * * * * * * * * * * * *

6613 GTGGAGTATCTCTTGAAAGCATGGGCGGAAAAGAAGGACCCCATGGGTTTTTCGTAcGATACCCGATGCTTCGACTCAACCGTCACcGAGAGAGACATCA 6712

V E Y L L K A W A E K K D P M G F S Y D T R C F D S T V T E R D I R

G V S L E S M G G K E G P H G F F V R Y P M L R L N R H R E R H Q

6720 6730 6740 6750 6760 6770 6780 6790 6800 6810

* * * * * * * * * * * * * * * * * * * *

6713 GGACCGAGGAGTCCATATACCAGGCCTGCTCCCTGCCCGAGGAGGCCCGCACTGCCATACACTCGCTGACcGAGAGACTTTACGTAGGAGGGCCCATGTT 6812

T E E S I Y Q A C S L P E E A R T A I H S L T E R L Y V G G P M F

D R G V H I P G L L P A R G G P H C H T L A D R E T L R R R A H V

6820 6830 6840 6850 6860 6870 6880 6890 6900 6910

* * * * * * * * * * * * * * * * * * * *

6813 CAACAGCAAGGGTCAAACCTGCGGTTACAGACGTTGCCGCGCCAGCGGGGTGCTAACCACcAGCATGGGcAACACCATCACATGCTATGTGAAAGCCCTA 6912

N S K G Q T C G Y R R C R A S G V L T T S M G N T I T C Y V K A L

Q Q Q G S N L R L Q T L P R Q R G A N H Q H G Q H H H M L C E S P S

6920 6930 6940 6950 6960 6970 6980 6990 7000 7010

* * * * * * * * * * * * * * * * * * * *

6913 GCGGCCTGCAAGGCTGCGGGGATAGTTGCGCCCACAATGCTGGTATGCGGCGAcGACCTAGTAGTCATCTCAGAAAGCCAGGGGACcGAGGAGGACGAGC 7012

A A C K A A G I V A P T M L V C G D D L V V I S E S Q G T E E D E R

G L Q G C G D S C A H N A G M R R R P S S H L R K P G D R G G R A

7020 7030 7040 7050 7060 7070 7080 7090 7100 7110

* * * * * * * * * * * * * * * * * * * *

7013 GGAACCTGAGAGCCTTCACGGAGGCCATGACCAGGTACTCTGCCCCTCCTGGcGATCCCCCCAGACCGGAATAcGACCTGGAGCTAATAACATCCTGTTC 7112

N L R A F T E A M T R Y S A P P G D P P R P E Y D L E L I T S C S

E P E S L H G G H D Q V L C P S W R S P Q T G I R P G A N N I L F

7120 7130 7140 7150 7160 7170 7180 7190 7200 7210

* * * * * * * * * * * * * * * * * * * *

7113 CTCAAATGTGTCTGTGGCGTTGGGCCCGCGGGGCCGCCGCAGATACTACCTGACCAGAGACCCAACCACTCCACTCGCCCGGGCTGCCTGGGAAACAGTg 7212

S N V S V A L G P R G R R R Y Y L T R D P T T P L A R A A W E T V

L K C V C G V G P A G P P Q I L P D Q R P N H S T R P G C L G N S E

7220 7230 7240 7250 7260 7270 7280 7290 7300 7310

* * * * * * * * * * * * * * * * * * * *

7213 AGACACTCCCCTATCAATTCATGGCTGGGAAACATCATCCAGTATGCTCCAACCATATGGGTTCGCATGGTCCTAATGACACACTTCTTCTCCATTCTCA 7312

R H S P I N S W L G N I I Q Y A P T I W V R M V L M T H F F S I L M

T L P Y Q F M A G K H H P V C S N H M G S H G P N D T L L L H S H

7320 7330 7340 7350 7360 7370 7380 7390 7400 7410

* * * * * * * * * * * * * * * * * * * *

7313 TGGTCCAAGACACCCTGGACCAGAACCTCAACTTcGAGATGTATGGATCAGTATACTCCGTGAATCCTTTGGACCTTCCAGCCATAATcGAGAGGTTACA 7412

V Q D T L D Q N L N F E M Y G S V Y S V N P L D L P A I I E R L H

G P R H P G P E P Q L R D V W I S I L R E S F G P S S H N R E V T

7420 7430 7440 7450 7460 7470 7480 7490 7500 7510

* * * * * * * * * * * * * * * * * * * *

7413 CGGGCTgGACGCCTTTTCTATGCACACATACTCTCACCACGAACTGACGCGGGTGGCTTCAGCCCTCAGAAAACTTGGGGCGCCACCCCTCAGGGTGTGG 7512

G L D A F S M H T Y S H H E L T R V A S A L R K L G A P P L R V W

R A G R L F Y A H I L S P R T D A G G F S P Q K T W G A T P Q G V E

7520 7530 7540 7550 7560 7570 7580 7590 7600 7610

* * * * * * * * * * * * * * * * * * * *

7513 AAGAGTCGGGCTCGCGCAGTCAGGGCGTCCCTCATCTCCCGTGGAGGGAAAGCGGCCGTTTGCGGCCGATATCTCTTCAATTGGGCGGTGAAGACCAAGC 7612

K S R A R A V R A S L I S R G G K A A V C G R Y L F N W A V K T K L

E S G S R S Q G V P H L P W R E S G R L R P I S L Q L G G E D Q A

7620 7630 7640 7650 7660 7670 7680 7690 7700 7710

* * * * * * * * * * * * * * * * * * * *

7613 TCAAACTCACTCCATTGCCGGAGGCGCGCCTACTGGACTTATCCAGTTGGTTCACCGTCGGCGCCGGCGGGGGCGACATTTTTCACAGCGTGTCGCGCGC 7712

K L T P L P E A R L L D L S S W F T V G A G G G D I F H S V S R A

Q T H S I A G G A P T G L I Q L V H R R R R R G R H F S Q R V A R

7720 7730 7740 7750 7760 7770 7780 7790 7800 7810

* * * * * * * * * * * * * * * * * * * *

7713 CCGACCCCGCTCATTACTCTTCGGCCTACTCCTACTTTTCGTAGGGGTAGGCCTCTTCCTACTCCCCGCTaGGTAGagcggcacacactaggtacactcc 7812

R P R S L L F G L L L L F V G V G L F L L P A R *

P T P L I T L R P T P T F R R G R P L P T P R *
