# Supplementary material for: Hepatitis C Virus RNA Replication Depends on Specific Cis- and Trans-Acting Activities of Viral Nonstructural Proteins
Source: PLoS Pathog. 2015 Apr 13;11(4):e1004817. doi: 10.1371/journal.ppat.1004817 (PMC4395149; doi:10.1371/journal.ppat.1004817)
Supplement: S3 Text — The wild-type JFH-1 NS3 gene (WT) was aligned to the NS3rc gene (RC), which contains recoded NS3 sequence downstream of SpeI. Mismatches are indicated by hash marks. The T269, D290, D291, and W501 residues are highlighted for emphasis. (DOCX) [file ppat.1004817.s008.docx]

**S3 Text. Sequence of NS3rc.** The wild-type JFH-1 NS3 gene (WT) was aligned to the NS3rc gene (RC), which contains recoded NS3 sequence downstream of SpeI. Mismatches are indicated by hash marks. The T269, D290, D291, and W501 residues are highlighted for emphasis.

* * * * * * * * * *

WT 1 gctcccatcactgcttatgcccagcaaacacgaggcctcctgggcgccatagtggtgagtatgacggggcgtgacaggacagaacaggccggggaagtcc 100

||||||||||||||||||||||||||||||||||||||||||||||||||||||||||||||||||||||||||||||||||||||||||||||||||||

RC 1 gctcccatcactgcttatgcccagcaaacacgaggcctcctgggcgccatagtggtgagtatgacggggcgtgacaggacagaacaggccggggaagtcc 100

A P I T A Y A Q Q T R G L L G A I V V S M T G R D R T E Q A G E V Q

* * * * * * * * * *

WT 101 aaatcctgtccacagtctctcagtccttcctcggaacaaccatctcgggggttttgtggactgtttaccacggagctggcaacaagactctagccggctt 200

||||||||||||||||||||||||||||||||||||||||||||||||||||||||||||||||||||||||||||||||||||||||||||||||||||

RC 101 aaatcctgtccacagtctctcagtccttcctcggaacaaccatctcgggggttttgtggactgtttaccacggagctggcaacaagactctagccggctt 200

I L S T V S Q S F L G T T I S G V L W T V Y H G A G N K T L A G L

* * * * * * * * * *

WT 201 acggggtccggtcacgcagatgtactcgagtgctgagggggacttggtaggctggcccagcccccctgggaccaagtctttggagccgtgcaagtgtgga 300

||||||||||||||||||||||||||||||||||||||||||||||||||||||||||||||||||||||||||||||||||||||||||||||||||||

RC 201 acggggtccggtcacgcagatgtactcgagtgctgagggggacttggtaggctggcccagcccccctgggaccaagtctttggagccgtgcaagtgtgga 300

R G P V T Q M Y S S A E G D L V G W P S P P G T K S L E P C K C G

* * * * * * * * * *

WT 301 gccgtcgacctatatctggtcacgcggaacgctgatgtcatcccggctcggagacgcggggacaagcggggagcattgctctccccgagacccatttcga 400

||||||||||||||||||||||||||||||||||||||||||||||||||||||||||||||||||||||||||||||||||||||||||||||||||||

301 gccgtcgacctatatctggtcacgcggaacgctgatgtcatcccggctcggagacgcggggacaagcggggagcattgctctccccgagacccatttcga 400

RC A V D L Y L V T R N A D V I P A R R R G D K R G A L L S P R P I S T

* * * * * * * * * *

WT 401 ccttgaaggggtcctcgggggggccggtgctctgccctaggggccacgtcgttgggctcttccgagcagctgtgtgctctcggggcgtggccaaatccat 500

||||||||||||||||||||||||||||||||||||||||||||||||||||||||||||||||||||||||||||||||||||||||||||||||||||

RC 401 ccttgaaggggtcctcgggggggccggtgctctgccctaggggccacgtcgttgggctcttccgagcagctgtgtgctctcggggcgtggccaaatccat 500

L K G S S G G P V L C P R G H V V G L F R A A V C S R G V A K S I

* * * * * * * * * *

WT 501 cgatttcatccccgttgagacactcgacgttgttacaaggtctcccactttcagtgacaacagcacgccaccggctgtgccccagacctatcaggtcggg 600

||||||||||||||||||||||||||||||||||||||||||||||||||||||||||||||||||||||||||||||||||||||||||||||||||||

RC 501 cgatttcatccccgttgagacactcgacgttgttacaaggtctcccactttcagtgacaacagcacgccaccggctgtgccccagacctatcaggtcggg 600

D F I P V E T L D V V T R S P T F S D N S T P P A V P Q T Y Q V G

* * * * * * * -SpeI- * *

WT 601 tacttgcatgctccaactggcagtggaaagagcaccaaggtccctgtcgcgtatgccgcccaggggtacaaagtactagtgcttaacccctcggtagctg 700

|||||||||||||||||||||||||||||||||||||||||||||||||||||||||||||||||||||||||||||||||||#||||||###||#||#|

RC 601 tacttgcatgctccaactggcagtggaaagagcaccaaggtccctgtcgcgtatgccgcccaggggtacaaagtactagtgctgaaccccagcgtggccg 700

Y L H A P T G S G K S T K V P V A Y A A Q G Y K V L V L N P S V A A

* * * * * * * * * *

WT 701 ccaccctggggtttggggcgtacctatccaaggcacatggcatcaatcccaacattaggactggagtcaggaccgtgatgaccggggaggccatcacgta 800

||||#|||||#|||||#||#|||||###||||||#||#||||||||#||||||||##||||#||#||#||#||||||||||||||#|||||||||||#||

RC 701 ccacactgggctttggcgcctacctgagcaaggcccacggcatcaaccccaacatccggaccggcgtgagaaccgtgatgaccggcgaggccatcaccta 800

T L G F G A Y L S K A H G I N P N I R T G V R T V M T G E A I T Y

* * * * * * * * * *

WT 801 ctccACAtatggcaaatttctcgccgatgggggctgcgctagcggcgcctatgacatcatcatatgcGATGAAtgccacgctgtggatgctacctccatt 900

|##|||#||#|||||#|||||#||||||||#|||||#||#|||||||||||#|||||||||||#|||||#||#||||||||#||#||#||#||###|||#

RC 801 cagcACCtacggcaagtttctggccgatggcggctgtgccagcggcgcctacgacatcatcatctgcGACGAGtgccacgccgtcgacgccacaagcatc 900

S T Y G K F L A D G G C A S G A Y D I I I C D E C H A V D A T S I

269 290 291

* * * * * * * * * *

WT 901 ctcggcatcggaacggtccttgatcaagcagagacagccggggtcagactaactgtgctggctacggccacaccccccgggtcagtgacaaccccccatc 1000

||#||||||||#||#||#||#||#||#||#||#||#||#||#||#|||||#||#||||||||#||#||#||#||#||#||####|||||#||||||||#|

901 ctgggcatcggcaccgtgctggaccaggccgaaaccgctggcgtgagactgaccgtgctggccaccgctaccccacctggcagcgtgaccaccccccacc 1000

RC L G I G T V L D Q A E T A G V R L T V L A T A T P P G S V T T P H P

* * * * * * * * * *

1001 ccgatatagaagaggtaggcctcgggcgggagggtgagatccccttctatgggagggcgattcccctatcctgcatcaagggagggagacacctgatttt 1100

WT ||||#||#||#||#||#|||||#||##|#|||||#||||||||||||||#||#||#||#||#|||||###||||||||||||#||#|||||||||||#||

1001 ccgacatcgaggaagtgggcctgggcagagagggcgagatccccttctacggcagagccatccccctgagctgcatcaagggcggcagacacctgatctt 1100

RC D I E E V G L G R E G E I P F Y G R A I P L S C I K G G R H L I F

* * * * * * * * * *

WT1101 ctgccactcaaagaaaaagtgtgacgagctcgcggcggcccttcggggcatgggcttgaatgccgtggcatactatagagggttggacgtctccataata 1200

#||||||###|||||#|||||#||||||||#||#||#|||||##|#|||||||||#||||#||||||||#|||||#|||||##|||||||#|||||#||#

RC1101 ttgccacagcaagaagaagtgcgacgagctggccgctgccctgagaggcatgggcctgaacgccgtggcctactacagaggcctggacgtgtccatcatc 1200

C H S K K K C D E L A A A L R G M G L N A V A Y Y R G L D V S I I

* * * * * * * * * *

WT1201 ccagctcagggagatgtggtggtcgtcgccaccgacgccctcatgacggggtacactggagactttgactccgtgatcgactgcaatgtagcggtcaccc 1300

||#||#|||||#||#|||||#||||||||||||||#|||||#|||||#||#|||||#||#|||||#|||##|||||||||||||||#||#||#||#||||

RC1201 cctgcccagggcgacgtggtcgtcgtcgccaccgatgccctgatgacaggctacaccggcgacttcgacagcgtgatcgactgcaacgtggccgtgaccc 1300

P A Q G D V V V V A T D A L M T G Y T G D F D S V I D C N V A V T Q

* * * * * * * * * *

WT1301 aagctgtcgacttcagcctggaccccaccttcactataaccacacagactgtcccacaagacgctgtctcacgcagtcagcgccgcgggcgcacaggtag 1400

|#||#||#|||||||||||||||||#||||||||#||#|||||#|||||#||#|||||#|||||#||#####|#||#|||||##|#||##|#|||||#||

RC1301 aggccgtggacttcagcctggaccctaccttcaccatcaccacccagaccgtgccacaggacgccgtgagcagaagccagcggagaggcagaacaggcag 1400

A V D F S L D P T F T I T T Q T V P Q D A V S R S Q R R G R T G R

* * * * * * * * * *

WT1401 aggaagacagggcacttataggtatgtttccactggtgaacgagcctcaggaatgtttgacagtgtagtgctttgtgagtgctacgacgcaggggctgcg 1500

|||##|#||||||||#||#||#||#||#|||||#||#||##|#|||###||#|||||#|||###||#|||||#||#|||||||||||#||#||#|||||#

RC1401 aggccggcagggcacctacagatacgtgtccaccggcgagagggccagcggcatgttcgactccgtggtgctgtgcgagtgctacgatgccggcgctgct 1500

G R Q G T Y R Y V S T G E R A S G M F D S V V L C E C Y D A G A A

* * * * * * * * * *

WT1501 TGGtacgatctcacaccagcggagaccaccgtcaggcttagagcgtatttcaacacgcccggcctacccgtgtgtcaagaccatcttgaattttgggagg 1600

|||||||||||#||#||#||#|||||#|||||##||||##|#||#||#||||||||#||#|||||#|||||||||||#|||||#||#|||||#|||||||

RC1501 TGGtacgatctgacccctgccgagacaaccgtgcggctgcgggcctacttcaacacccctggcctgcccgtgtgtcaggaccacctggaattctgggagg 1600

W Y D L T P A E T T V R L R A Y F N T P G L P V C Q D H L E F W E A

501

* * * * * * * * * *

WT1601 cagttttcaccggcctcacacacatagacgcccacttcctctcccaaacaaagcaagcgggggagaacttcgcgtacctagtagcctaccaagctacggt 1700

|#||#|||||||||||#||#|||||#|||||||||||#||###|||#||#|||||#||#||||||||||||||#|||||#||#|||||#||#||#||#||

RC1601 ccgtgttcaccggcctgacccacatcgacgcccactttctgagccagaccaagcaggcaggggagaacttcgcctacctggtcgcctatcaggcaacagt 1700

V F T G L T H I D A H F L S Q T K Q A G E N F A Y L V A Y Q A T V

* * * * * * * * * *

WT1701 gtgcgccagagccaaggcccctcccccgtcctgggacgccatgtggaagtgcctggcccgactcaagcctacgcttgcgggccccacacctctcctgtac 1800

|||||||||||||||#|||||||||||###||||||||||||||||||||||||||||#||||#||||||||#||#||#||#||#||#||#||#||||||

RC1701 gtgcgccagagccaaagcccctccccctagctgggacgccatgtggaagtgcctggccagactgaagcctaccctggccggacctacccccctgctgtac 1800

C A R A K A P P P S W D A M W K C L A R L K P T L A G P T P L L Y

* * * * * * * * *

WT1801 cgtttgggccctattaccaatgaggtcaccctcacacaccctgggacgaagtacatcgccacatgcatgcaagctgaccttgaggtcatgacc 1893

#|##|||||||#||#|||||#||#||#|||||#||#|||||#||#||#|||||#||||||||#||#|||||#||#|||||#||#||#||||||

RC1801 agactgggccccatcaccaacgaagtgaccctgacccaccccggcaccaagtatatcgccacctgtatgcaggccgacctggaagtgatgacc 1893

R L G P I T N E V T L T H P G T K Y I A T C M Q A D L E V M T
